# Thermal Decomposition of Date Seed/Polypropylene Homopolymer: Machine Learning CDNN, Kinetics, and Thermodynamics

**DOI:** 10.3390/polym17030307

**Published:** 2025-01-23

**Authors:** Zaid Abdulhamid Alhulaybi Albin Zaid, Abdulrazak Jinadu Otaru

**Affiliations:** Department of Chemical Engineering, College of Engineering, King Faisal University, Al Ahsa 31982, Saudi Arabia

**Keywords:** thermal decomposition, date seed, polypropylene homopolymer, bio-composite, machine learning CDNN, kinetics and thermodynamics

## Abstract

The buildup of abandoned plastics in the environment and the need to optimize agricultural waste utilization have garnered scrutiny from environmental organizations and policymakers globally. This study presents an assessment of the thermal decomposition of date seeds (DS), polypropylene homopolymer (PP), and their composites (DS/PP) through experimental measurements, machine learning convolutional deep neural networks (CDNN), and kinetic and thermodynamic analyses. The experimental measurements involved the pyrolysis and co-pyrolysis of these materials in a nitrogen-filled thermogravimetric analyzer (TGA), investigating degradation temperatures between 25 and 600 °C with heating rates of 10, 20, and 40 °C.min^−1^. These measurements revealed a two-stage process for the bio-composites and a decrease in the thermal stability of pure PP due to the moisture, hemicellulose, and cellulose content of the DS material. By utilizing machine learning CDNN, algorithms and frameworks were developed, providing responses that closely matched ($R^{2}\sim 0.942$) the experimental data. After various modelling modifications, adjustments, and regularization techniques, a framework comprising four hidden neurons was determined to be most effective. Furthermore, the analysis revealed that temperature was the most influential parameter affecting the thermal decomposition process. Kinetic and thermodynamic analyses were performed using the Coats–Redfern and general Arrhenius model-fitting methods, as well as the Flynn–Wall–Ozawa and Kissinger–Akahira–Sunose model-free approaches. The first-order reaction mechanism was identified as the most appropriate compared to the second and third order F-Series solid-state reaction mechanisms. The overall activation energy values were estimated at 51.471, 51.221, 156.080, and 153.767 kJ·mol^−1^ for the respective kinetic models. Additionally, the kinetic compensation effect showed an exponential increase in the pre-exponential factor with increasing activation energy values, and the estimated thermodynamic parameters indicated that the process is endothermic, non-spontaneous, and less disordered.

## 1. Introduction

The 2030 United Nations Sustainable Development Goals (UN-SDGs) prioritize the prevention and significant reduction in aquatic (Goal 14) and land (Goal 15) pollution, as well as the doubling of global energy utilization and efficiency improvements (Goal 7) [1]. The escalating production of plastics by petrochemical industries, coupled with policymakers’ growing support for agricultural expansion and food security, has led to an increase in agricultural and plastic waste polluting the environment. While agricultural waste is known to be biodegradable, most plastic wastes are non-biodegradable, and it may take several decades for the remaining few to fully decompose in landfills [2]. According to a report by Vuppaladadiyam et al. [3], plastic production is projected to reach 25,000 MMT by 2040 if the accumulation of discarded plastic waste is not controlled. This mounting plastic waste could exacerbate current ecological and environmental issues caused by existing plastics in the environment. The current global plastic recycling rate is less than 10% [3], mainly due to the rising energy costs associated with the thermal degradation of such materials. Consequently, scientists and engineers face the formidable challenge of developing an energy-efficient roadmap for this process while also reducing the abundance of waste materials that litter our environment.

Previous research studies have primarily focused on the energy demand and time required for the complete decomposition of plastics, biomass, and biopolymer blends through pyrolysis. Bahl et al. [4] noted that the enzymatic biodegradation of plastics by microorganisms facilitates the breakdown of polymers into smaller monomers. This approach has provided insights into the kinetics, thermodynamics, operating conditions, and reaction mechanisms involved in these chemical processes. Researchers in this field have predominantly employed pyrolysis and co-pyrolysis methods in thermogravimetric analyzers (TGAs) to investigate the decomposition of discarded plastics and biomass wastes. Parameters such as temperature, time, heating rate, and material compositions have been evaluated. Uzoejinwa et al. [5] conducted a critical assessment and concluded that the co-pyrolysis of biomass and waste plastics offers greater benefits compared to the sole pyrolysis of biomass. This alternative approach could improve energy efficiency, reduce dependence on fossil fuels, and contribute to effective waste management. In a separate study, Çepelioğullar and Pütün [6] investigated the co-pyrolysis of biomass–plastic blends. The experiment involved specific ratios (1:1, *w*/*w*) of agricultural wastes, namely cotton stalk, sunflower residue, hazelnut shell, and arid land plant *Euphorbia rigida*. These waste materials were mixed with polyvinyl chloride (PVC) and polyethylene terephthalate (PET) in a TGA system, with a constant heating rate of 10 °C.min^−1^. The study [6] revealed that the structural differences in these materials significantly impact their pyro-degradation mechanisms. Moreover, the activation energy necessary to degrade the two plastics was found to be considerably higher than that of the biomass materials. This approach also resulted in notable variations in the mass loss of individual materials and their blends, indicating distinct differences in their thermo-kinetic behaviors.

The significance of the ratio of biomass to polymer has been shown to have a significant impact on the thermo-kinetic behavior of biomass-plastics. As a result, changes in reaction pathways and energy requirements for these processes have been observed. For example, a study by Chin et al. [7] investigated the thermal decomposition of rubber seed shell (RSS), high-density polyethylene (HDPE), and composites of both materials (HDPE/RSS) using non-isothermal TGA analysis with weight ratios ranging between 0.2 and 0.4. Various conditions of heating rates between 10 and 50 °C.min^−1^, and degradation temperatures between 323 and 1173 K, were selected for these different feedstock ratios. The study reported distinct pathways and differences in the amount of residual ash achieved under these varying process parameters. Furthermore, the study [7] provided estimated activation energy values for these materials using first-order reaction kinetics as follows: 46.94–63.21 kJ.mol^−1^ for RSS, 242.13–278.14 kJ.mol^−1^ for HDPE, and 49.14–83.11 kJ.mol^−1^ for HDPE/RSS samples. Xiang et al. [8] demonstrated that the thermal decomposition of rice straw (RS) or lignocellulosic biomass and linear low-density polyethylene (LLDPE) using a modified ZSM-5 catalyst is a more complex process than the pyrolysis of individual materials. The activation energy values calculated for the RS/LLDPE composite and RS alone were reported as 59.7 kJ·mol^−1^ and 79.61 kJ·mol^−1^, respectively, indicating the presence of synergistic interactions between the two materials. Singh et al. [9] conducted a TGA experimental study on the thermal decomposition of wheat straw (WS) and polyethylene (PE), employing the Kissinger–Akahira–Sunose (KAS), Flynn–Wall–Ozawa (FWO), and Starink’s (STK) iso-conventional model-free methods to estimate the kinetic and thermodynamic parameters of the materials. The activation energy value for WS reported in the study [9] was 164.33 kJ·mol^−1^, while a reduction in the activation energy of the polymer (220.79 kJ·mol^−1^) was observed for the composite (197.36 kJ·mol^−1^). Chen et al. [10] investigated the thermal decomposition of polyvinyl chloride (PVC), polyethylene (PE), and polypropylene (PP) plastics mixed with Paulownia wood (PW) using a TGA. The study [10] revealed that the blends exhibited higher residual char yields compared to pure plastic, indicating a decrease in the thermal stability of the plastics. A study on the isothermal combustion and kinetics of a blend of waste polystyrene plastics fuel (RPF) and wastewater sludge (WS) reported in [11] examined polymer-to-material weight ratios ranging from 0 to 1. The study found that increasing the proportion of plastics in the blends enhanced the thermal stability of the WS, with the estimated activation energy increasing from 12.43 to 58.6 kJ·mol^−1^.

While most research on the thermal decomposition of materials found in the literature is based on experimental methods, there has been a growing interest among energy and environmental experts in virtually replicating the behavior observed in these experiments. Recently, researchers have turned to machine learning tools to describe the degradation profiles of plastics and biomass. Specifically, in 2023, Potnuri et al. [12] examined the accuracy of a machine learning support vector machine (SVM) model in predicting the pyrolysis yield from microwave co-pyrolysis of biomass-plastics. Their study [12] showed that the SVM model demonstrated strong predictive capabilities, with coefficient of determination (R^2^) values of 0.91, 0.93, and 0.96 for biogas, biochar, and bio-oil yields, respectively. Song et al. [13] also found excellent performance in predicting the elemental distribution, yield, and degree of aromatization of biochar obtained from the thermal decomposition of biomass using the extreme gradient boosting (XGB) algorithm. In a study by Alhulaybi and Otaru [14], the potential of backpropagation deep neural networks (DNN) in predicting weight loss profiles resulting from the co-pyrolysis of *Phoenix dactylifera* L. (PD) and high-density polyethylene (HDPE) was explored. While the study [14] accurately predicted the resulting thermograms obtained from thermogravimetric analysis (TGA), with an overall residual error of 0.025, the developed model failed to account for the influence of blend ratio on the trained DNN-computed data. Furthermore, the application of machine learning DNN in another study [15] successfully predicted the weight loss and degradation temperature from the thermal decomposition of sewage sludge and peanut shell at low heating rates (5, 7, and 10 °C.min^−1^), resulting in the establishment of an ANN21 model that provided the best predictions within the range of experimental variability.

Several other researchers [16,17,18] have also implemented machine-learning tools for predicting the mass profiles of biomass-plastic behavior. However, the number of research studies utilizing these machine learning tools is considerably smaller compared to the extensive amount of experimental-based research reported in the literature within this field. Moreover, the escalating accumulation of agricultural and plastic wastes in the environment raises significant concerns. Policymakers and environmental organizations worldwide have persistently emphasized the need to convert waste into energy and wealth. Consequently, this study represents the first instance in which a combination of convolutional neural network (CNN) and deep neural network (DNN) machine learning tools were employed to predict the mass loss profiles resulting from the co-pyrolysis of date seed (DS) and polypropylene homopolymer (PP). Additionally, the research explores the application of 1st, 2nd, and 3rd order solid-state reaction mechanisms, as well as the model-fitting Coats–Redfern and generalized Arrhenius methods, to estimate the kinetic and thermodynamic parameters for DS, PP, and DS/PP at varying percentage ratios and heating rates of 10, 20, and 40 °C.min^−1^.

## 2. TGA Experimental Approach and Data

The experimental procedure utilized in this study was derived from similar procedures documented in previous studies [14,16,19,20] regarding the thermal decomposition of discarded date seed (DS) and waste polypropylene homopolymer (PS). The polypropylene homopolymer in question is the PP 520L type, which was procured from the Saudi Basic Industries Corporation (SABIC). SABIC developed this material for use in the production of tubular waste quenched blown film, which requires the addition of slip and anti-blocker additives [21]. Although the PP 520L has also been utilized in the processing of food and textile packaging, magazine covers, and garments, among others, the date seeds utilized in this study were obtained as part of discarded agricultural waste from the Al-Ahsa province in the Kingdom of Saudi Arabia.

Samples of agricultural waste (DS) and polypropylene homopolymer (PP) were collected, separated from unwanted materials, and dried at a temperature of 50 °C for over 12 h. These materials were then reduced to powdered forms and sieved to particle sizes ranging between 100 and 200 µm. Synthetic mixtures of these samples were created by adding a fraction of the DS to the PP material to form composites (DS/PP) with varying percentage ratios: 0, 25, 50, and 100%. Both pyrolysis and co-pyrolysis of these materials (DS, PP, and DS/PP) were conducted using a Mettler Toledo TGA/SDTA851e thermogravimetric analyzer (TGA) equipped with STARe V7.01 recording software. The heating chamber within the TGA system was made inert by introducing a 40 mL.min^−1^ flow of nitrogen gas ($N_{2}$), while the heating rates used for the TGA analysis were set at 10, 20, and 40 °C.min^−1^. It is worth noting that the TGA environment was made inert to prevent material loss due to oxidation (combustion) during the thermal decomposition process. Before the pyrolysis and co-pyrolysis operational studies, the DS, PP, and DS/PP blends were weighed. The degradation temperature ranged between 25 and 600 °C. The measured process operating conditions during the experimentation included heating rate (Q), degradation temperature (T), time (t), and percentage composition of the blends (${DS}_{C}$). After reaching the maximum degradation temperature of 600 °C, the TGA system was turned off, and the sample was allowed to cool before weighing. A total of twelve different experimental datasets were obtained for the varying degrees of the operating conditions, which were then analyzed. The data obtained from the TGA experiment were analyzed to estimate the derivative thermogravimetric (DTG) values, calculated as the ratio of the derivative of the weight loss to the derivative of temperature.

**Figure 1:**
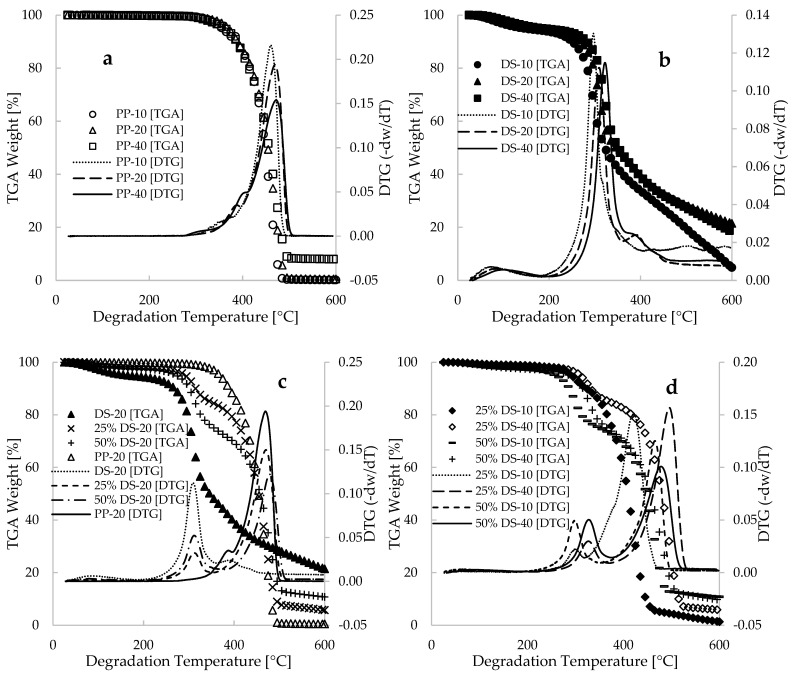
Plots depicting experimental TGA-DTG data against degradation temperature [°C] for (**a**) pure polypropylene [PP], (**b**) pure date seed [DS], (**c**) composites composed of DS/PP under condition of constant heating rate, and (**d**) composites subjected to varied heating rates.

Figure 1 illustrates the plots of percentage weight loss of samples (TGA) and the derivative thermogravimetric (DTG) data against degradation temperature (25–600 °C) for the pure (DS and PP) and blends or bio-composite (DS/PP) materials analyzed at heating rates of 10, 20, and 40 °C.min^−1^. Both the pure polypropylene homopolymer (Figure 1a) and the date seed (Figure 1b) exhibit a decomposition process that can be categorized into three stages: low, intermediate, and final (high) temperature decomposition. As shown in Figure 1a, the initiation or low-temperature decomposition for the PP sample takes place within a temperature range of 25 to 325 °C, constituting less than 2.5 wt% of the total mass at this stage for all the heating rates considered. This low weight can be attributed to the extremely low moisture content of the pure PP material, which is reportedly less than 0.2%, substantiated by the study [22]. Huang et al. [23] demonstrated that the low-temperature decomposition stage for polypropylene occurs at a degradation temperature below 300 °C. This stage is characterized by the dissociation of hydrogen radicals resulting from the loss of water molecules, changes in bond and dihedral angles, and elongation of atomic bonds. Consequently, this stage of degradation in Figure 1a can be described as amorphous [24], and a further increase in temperature could alter the polymer characteristics. Figure 1a indicates that the intermediate or decomposition stage of the PP sample was achieved at temperatures ranging between 325 and 495 °C. The thermal degradation stage was reported in [25,26] to be characterized by the rapid depolymerization of various larger molecules into smaller ones due to the scission or breakage of the C-C bonds, resulting in the polymorphic conversion from amorphous to semi-crystalline materials characterized by the cross-linking of molecules. Peterson et al. [27] described this polymorphic conversion as a random macromolecular main-chain scission or crack, which often leads to the release of free radicals that further decompose into several products through intermolecular and intramolecular hydrogen transfer reactions, resulting in the formation of smaller molecules known as monomers.

According to a report by Huang et al. [23], the pyrolysis of propylene during the intermediate stage of the process achieved degradation temperature values ranging from 277 to 477 °C. A TGA experiment conducted in [25] demonstrated that polypropylene degrades in a single intermediate step, beginning from 300 to 475 °C. In contrast, the same study [25] revealed that a TGA experiment conducted for a similar sample in the presence of air (combustion) resulted in the attainment of the intermediate decomposition step at 250 and 425 °C. Accordingly, thermo-oxidative decomposition shifts could lead to single or multi-step degradation, depending on the composition of the materials. In this study, the DTG data for the PP plastic strongly favor a single step degradation, with the intermediate region showing a slight change for thermogram obtained at 20 °C.min^−1^ of heating rate. The final decomposition stage of the PP material (Figure 1a) is achieved at a temperature beyond 495 °C and is largely characterized by a large amount of smaller or depolymerized molecules, also known as residual ash. The raw experimental data obtained for this study shows that the residual materials for the PP at heating rates of 10, 20, and 40 °C.min^−1^ are 0.111, 0.492, and 7.92 wt%, respectively. This low residual ash can be attributed to the extremely low or no-ash content associated with polypropylene [22]. It is important to note that the impact of varied heating rates on the thermal degradation of the selected grade of PP plastic is minimal (Figure 1a). This characteristic may be attributed to slight variations in the density of the selected PP material (0.895–0.92 g.cm^−3^ [28]), which result in minor density changes between the amorphous and crystalline phases of the material during thermal degradation. Additionally, this limited effect could be ascribed to the material’s composition, which may necessitate minimal differences in the heating rates employed to reveal discrepancies in mass loss profiles.

Figure 1b demonstrates that the thermal decomposition of the pure date seed (DS) powder proceeds in three stages under a nitrogen gas-filled (inert) environment. The figure reveals that at higher heating rates, the thermogram of the mass loss profile shifts towards a higher temperature compared to the profile at lower heating rates. This shift could be attributed to the shorter heating time required for complete decomposition at higher heating rates. At relatively low degradation temperatures, typically ranging from 25 to 265 °C, the mass loss of the pure DS sample accounts for approximately 12.5 wt% of the initial sample. This substantial mass loss can be attributed to the high moisture content and volatile organic matter present in the DS sample. Bouaziz et al. [29] conducted an experimental study on the physicochemical properties of powdered Saudi Arabia date seeds, reporting moisture contents ranging from 4.76 to 8.02%. Additionally, Bouhlali et al. [30] reported moisture contents between 4.6 and 8.3% for date seeds obtained from three different regions in Morocco. This indicates that the loss of moisture content during the dehydration stage (low temperature) of DS decomposition contributes significantly to the mass loss of the material. Figure 1b also indicates that the intermediate temperature range for DS decomposition is 265 to 395 °C, accounting for over 60 wt% mass loss. This stage can be attributed to the volatilization of extractive compounds, as well as the decomposition of cellulose, hemicellulose, and lignin components in the DS material [31,32]. Bouaziz et al. [29] determined that the cellulose, hemicellulose, lignin, and ash contents of various Saudi Arabia date seeds fall within the ranges of 26.60–33.92 wt%, 31.97–42.30 wt%, 21.20–24.06 wt%, and 0.78–2.32 wt%, respectively. The high ash content of the DS material may contribute to the increased residual weight observed during DS degradation (Figure 1b), in comparison to the lower residual weight of the pure PP shown in Figure 1a.

Since the decomposition stages of pure DS and pure PP occur at different degradation temperatures, it is important to note that the thermal degradation of the bio-composite or blends (DS/PP) is likely to occur at different temperatures, indicating different reaction mechanisms. Figure 1c illustrates that the addition of DS to PP alters the degradation process, transforming the one-step process observed in pure PP at its intermediate stage into a two-step process. This finding is consistent with a study by [22], which demonstrated a two-step process for the co-pyrolysis of walnut shells and polypropylene. This suggests that, under a constant heating rate of 20 °C.min^−1^, the high moisture content of the DS sample leads to early degradation of the DS/PP bio-composite, resulting in reduced thermal stability and a shift in the thermogram towards lower temperatures, compared to the mass loss profile of pure PP with high thermal stability. For instance, in the lower temperature range of approximately 25 to 285 °C, mass losses of approximately 7.0 and 9.0 wt% were estimated for DS additions of 25% and 50% in the bio-composite, respectively. Beyond this range, the presence of cellulose and hemicellulose components in the DS sample contribute significantly to the mass loss observed during the co-pyrolysis process of DS/PP samples.

The DTG plots presented in Figure 1 elucidate the characteristic behavior of the experimentally measured data concerning varying compositions and heating rates. Higher DTG peaks indicate a greater loss of derivative weight at specific degradation temperatures. In Figure 1a, the TGA data of PP plastics with overlaps at different heating rates are clearly more supported by the DTG curves, shifting peaks to high temperatures with increasing heating rates, and reduction in derivative weight loss. The two broad peaks observed in the 25% DS-20 and 50% DS-20 samples can be attributed, initially, to the loss of hemicellulose and cellulose in the biomass, followed by the degradation of the plastic content in the composites at elevated temperatures. These figures demonstrate that an increase in biomass composition results in a greater weight loss at temperature minima, while an increase in the PP plastic content corresponds to a higher weight loss at temperature maxima. For instance, in the case of the 50% DS-20 sample, a more significant loss of biomass is observed at a broad peak of approximately 314 °C compared to the weight loss observed in the 25% DS-20 sample at the same temperature. Conversely, at higher temperatures, a greater quantity of the PP plastic materials is lost in the 25% DS-20 sample compared to the 50% DS-20 sample. Furthermore, the DTG curves also shift to higher temperatures with increasing heating rates, suggesting enhanced thermal stability of materials undergoing thermal degradation at elevated heating rates. In a related study conducted by Turku et al. [33], an investigation was carried out on the thermal degradation of polypropylene and wood fiber materials. The findings of this study suggested that when higher proportions of polypropylene (PP) were present in the bio-composite, there was a greater potential for PP particles to cover the DS material. As a result, this led to delayed degradation and increased thermal stability due to the low thermal conductivity of DS sample. Figure 1c,d support this claim and demonstrate that increasing the DS content from 25% to 50% in the bio-composite results in reduced weight of the PP material and decomposition mechanisms that can be attributed to the increasing cellulose and hemicellulose components of the biomass material. Furthermore, these figures indicate that an increase in the biomass (DS) content within the bio-composite results in elevated lignin composition, which consistently impedes degradation at elevated temperatures, typically exceeding 500 °C.

## 3. Machine Learning CDNN Analysis of Data

The term CDNN, as used in this study, is an acronym for convolutional deep neural networks. It encompasses a combination of machine learning techniques, specifically the fusion of convolutional neural networks (CNN) and deep neural networks (DNN). While DNN has been extensively utilized in the analysis of experimental or real-world data, CNN is a distinct type of artificial neural network primarily designed for image recognition and processing [34]. However, recent research developments have expanded the application of CNN to various other domains of data analysis, notably in the prediction of properties of polymeric materials [35,36,37].

**Figure 2:**
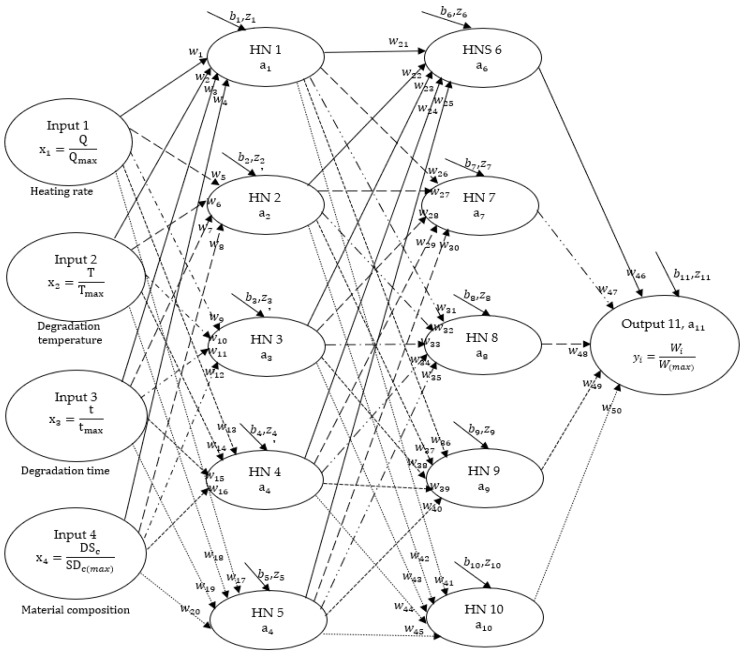
Illustration of a machine learning deep neural network (DNN) framework displaying the standard configuration of input, hidden, and output layers, each composed of 10 hidden neurons, denoted as 10 HNs (see Supplementary Information).

In this study, the backpropagation method of deep neural networks (DNN) was initially employed to train selected experimental datasets on the thermal decomposition of DS, PP, and DS/PP blends. Subsequently, a combination of convolutional neural networks (CNN) and DNN, referred to as CDNN was applied. Figure 2 illustrates a proposed DNN framework, which consists of four inputs, ten hidden neurons (HNS), and an output connected by synaptic weights ($w_{i}$) and biases ($b_{k}$). The ten HNS in the framework are evenly distributed into two layers, forming a DNN(2[5,5] HNS) network. The input parameters for the experiment are the heating rate (Q), degradation temperature (T), degradation time (t), and the fraction compositions of DS in the composite (${DS}_{c}$). The output parameter is the mass loss ($W_{i}$) resulting from the decomposition of the material at varying temperatures. The general artificial neural network (ANN) equation, combined with the sigmoid activation function (Equations (1)–(3)), was used to develop learning algorithms based on the backpropagation approach, using the framework shown in Figure 2. For more detailed information on the development of machine learning algorithms utilizing the backpropagation technique, please refer to previous research conducted by this group [14,19,38,39].

Equation (1) presents a mathematical expression that demonstrates a linear relationship between the sum weight (Z) and the synaptic weight ($w_{i}$), input function ($x_{i}$), and biases ($b_{k}$). Equation (2) represents the Sigmoid activation function (a) as a test function of the sum weight (Z). This activation function was chosen due to its suitability in training log-transformed data, typically ranging between 0 and 1 [40]. Consequently, both the input and output experimental datasets used for training were logarithmically transformed to values between 0 and 1 by dividing them with their respective maximum values achievable within the TGA utilized for acquiring the experimental data. For example, the maximum values for the heating rate ($Q_{max}$), degradation temperature ($T_{max}$), degradation time ($t_{max}$), and DS composition in the blends (${SD}_{c(max)}$) were 100 °C.min^−1^, 1000 °C, 7200 s, and 1.00, respectively. Equation (3) represents the mathematical expression for the overall cost function (C) or the residual error between the experimental (y) and predicted (a) weight loss of the sample during degradation.

(1) $$Z=\sum_{i}w_{i}\cdot x_{i}+b_{k}$$

(2) $$a=\sigma^{\prime }[z]=\frac{1}{1+e^{-z}}$$

(3) $$C={\left(y-a\right)}^{2}$$

The significance of experimental datasets and individual data points in machine learning applications cannot be overstated [41], as they make a substantial contribution to the output of the models. In this study, a total of 12 experimental datasets, comprising 708 data points, were selected from the raw experimental data (24,152 data points) for training purposes. The 708 data points were carefully chosen using a temperature spacing of 10 °C, ensuring equal intervals, from the acquired TGA experiment data. The TGA experiment involved degradation temperatures ranging from 25 to 600 °C. The training of these data points was carried out using a written code in MS Visual Basics for Applications (VBA). The formulated algorithms were solved for a specific duration of time. Initially, the learning rate (L) was set to 5.0, and the training datasets underwent the learning algorithms for one second. Subsequently, the learning process was accelerated by setting the time to zero seconds. The overall cost function (C), true error, and number of passes required for the training datasets to complete the learning algorithms (epochs) were recorded at regular intervals throughout the learning process. The objective of this approach was to obtain computed data (synaptic weights and biases) necessary for the accurate prediction of the mass profile loss observed in the TGA experimentation. This goal could be achieved by systematically training the experimental datasets and reducing the overall cost function or true errors to nearly zero. This signifies the point of convergence between predictions and experimental results [39].

**Figure 3:**
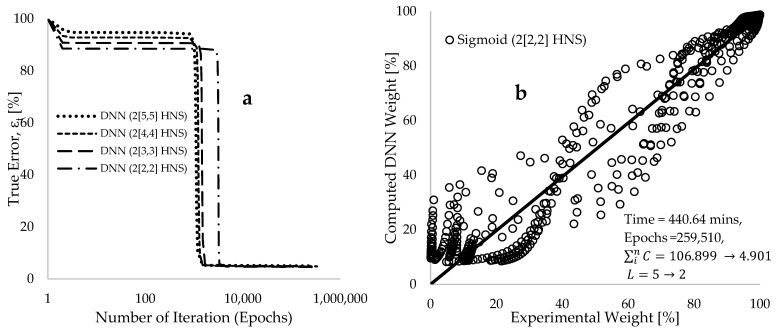
Computed data from deep neural network (DNN) modelling reveals (**a**) plots depicting the true error against epochs for 10, 8, 6, and 4 hidden neuron layers (HNS), and (**b**) plots illustrating the DNN-computed weight percentages compared to the corresponding experimental data percentages.

Figure 3a depicts the relationship between true error ($\varepsilon_{t}$) and the number of iterations (Epochs) for four modified deep neural network (DNN) frameworks. These frameworks, namely DNN (2[5,5]HNS), DNN (2[4,4]HNS), DNN (2[3,3]HNS), and DNS (2[2,2]HNS), consist of artificial neural networks (ANN) with two layers and varying numbers of hidden neurons (HNS), specifically 10, 8, 6, and 4. The figure illustrates that training the DNN using the initially constructed framework with 10 HNS led to a reduction in the percentage of true error from 100% to 4.96% over approximately 291,769 epochs. It is important to note that this 4.96% true error represents a significant deviation between the model and experimental values. Moreover, it is anticipated that the generalization of this model to future data about the mass loss profile of such materials may be unsuccessful, indicating underfitting. To mitigate the impact of underfitting, modelling regularization was introduced to encourage the learning of more generalized patterns by the model. Additionally, modifications were made to the initial DNN (2[5,5]HNS) framework, including reducing the number of HNS to 8, 6, and 4, and adjusting the learning rate from 5 to 1 during the training process. Subsequently, each reduced framework was independently trained. The objective of this approach is to simplify the learning algorithms, thereby reducing the size of the model (pruning), increasing learning speed, and improving efficiency. It is noteworthy that a learning rate of 5.0 was initially chosen for training based on preliminary cross-validation, aiming to strike a balance between modelling convergence, stability, and training speed. Preliminary observations indicated that a higher learning rate carried the risk of overshooting the optimal solution, while a lower learning rate resulted in slower training. By setting the initial learning rate at 5.0 and gradually decreasing it to 1, efficient learning of the formulated algorithms with the experimental datasets could be ensured, maintaining stability and avoiding overshooting. Figure 3a demonstrates some improvements with this approach during the initial learning cycle, resulting in a further decrease in the true error to 4.56 for the DNN (2[2,2]HNS) framework. Figure 3b presents plots of the predicted response using the DNN (2[2,2]HNS) framework against the experimental data of TGA weight loss for all the 708 selected data points. Despite the use of a significant number of epochs (259,510) and over 440 min of modelling time, Figure 3b indicates that the DNN model performed poorly in accurately capturing the behavior characterized by the experiments. This substantial deviation between the experiment and modelling data could potentially be reduced by employing convolutional neural networks (CNNs) to extract crucial information from the experimental datasets for DNN training.

Machine learning convolutional neural networks (CNN) were applied to the 12 selected experimental datasets (totaling 708 data points) for log-normalizing these datasets. This method involves passing individual datasets through a multi-layer perceptron (MLP) consisting of a series of filters/kernels to obtain a vector [42]. The objective is to enable the individual dataset to adaptively learn latitudinal ordering or power structures through backpropagation, utilizing various filters such as convolutional layers, padding, sliding, etc. [34,43]. For a one-dimensional sequence, the mathematical equation describing the relationship between the input sequence ($X\in R^{n}$) and a convolutional filter ($K\in R^{2m+1}$) is mathematically expressed by Equation (4), as provided in [43].

(4) $$\mathrm{Output}\;\mathrm{vector}:\;h\left(t\right)=\left(x*t\right)=\sum_{\tau =-m}^{m}x\left(t-\tau \right)\cdot k(\tau )\;$$

where 2m+1 represents the convolutional operator, which exclusively supports odd-length filters, x denotes the input vector, k represents the filter or kernel, and t represents the time shift in the input resulting from a corresponding shift in the output convolution. This phenomenon is commonly referred to as translation equivariance in image processing [42].

**Figure 4:**
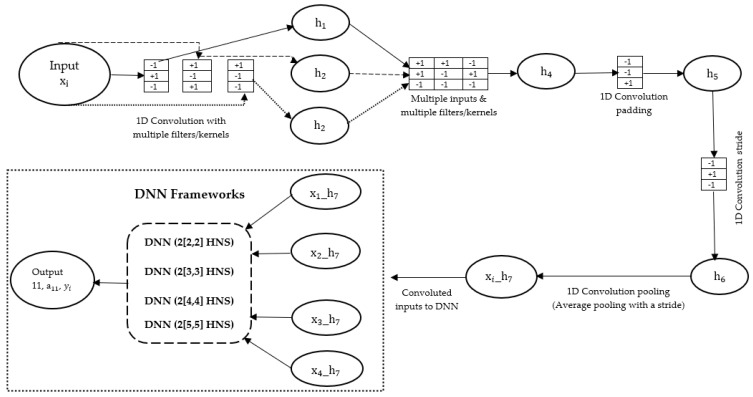
An illustration depicting the utilization of convolutional deep neural networks (CDNN) in the filtration and training processes of specific experimental datasets.

Figure 4 illustrates the utilization of a multi-layer perceptron (MLP) within the CDNN for log normalization and training of the experimental datasets. The initial step involved passing the input vector through three distinct filters, resulting in the estimation of three output vectors: $h_{1}$, $h_{2}$, and $h_{3}$. Subsequently, these output vectors underwent a filtering process (employing multiple inputs and multiple kernels) to obtain a single output vector. However, it is important to note that the effects imposed by the boundaries of each vector were not considered, leading to a reduction in the dimensionality of the output vectors. To address this issue, a one-dimensional padding of the output vector ($h_{4}$) was performed by filling the two boundaries with zero (0) and subsequently applying a filter length (−1/2) around the data to preserve the dimensionality. This padding technique ensured an equal number of data points (55) between the input ($h_{4}$) and output ($h_{5}$) vectors. Subsequently, a two-step stride was applied to the output vector ($h_{5}$) to further reduce its dimensionality for the final classification ($h_{6}$). Finally, a deterministic function of average pooling was applied to the output vector ($h_{6}$) to achieve a reduction in the final classification ($x_{i}\_h_{7}$), which yielded values ranging between 0 and 1.00.

**Figure 5:**
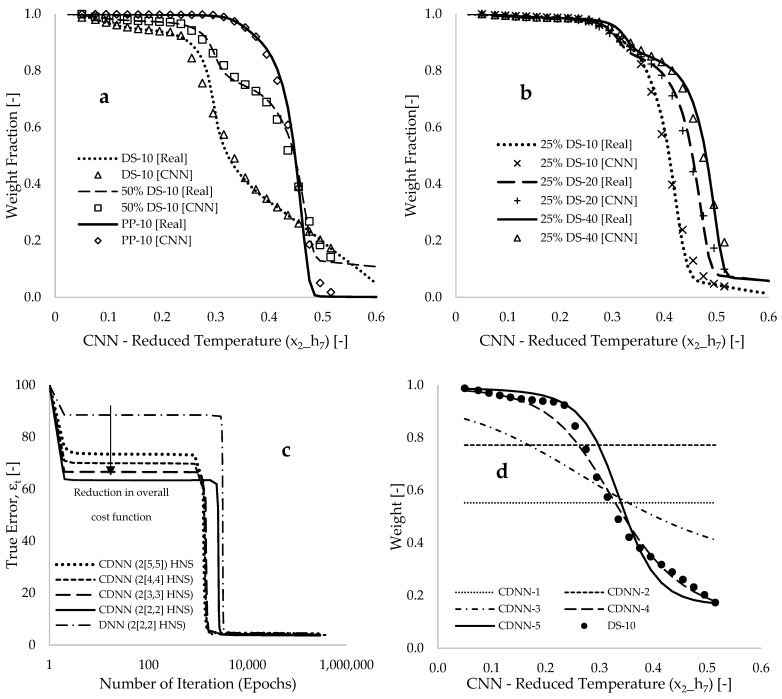
Plots of real and convoluted (CNN) weight fraction [-] against reduced degradation temperature [-] for the pure DS, PP, and composites for (**a**) a constant heating rate of 10 °C.min^−1^ and (**b**) constant material composition. Data from CDNN and DNN modelling showing (**c**) plots of true error against epochs computed for 10, 8, 6, and 4 HNS and (**d**) plots of experimental and CDNN computed weight [%] against CNN—reduced temperature [-] for the DS-10 sample at selected stages of computational trainings.

Figure 5 illustrates that the reduced final classification ($x_{i}\_h_{7}$) serves as an input to multiple DNN frameworks by learning the formulated learning algorithms of each. Figure 5a,b display plots of the reduced final classification and experimental data of mass loss during degradation against reduced degradation temperature ($x_{2}\_h_{7}$). A total of 288 data points were obtained after applying CNN to the datasets, representing approximately 40% of the initial input data points of 708. Figure 5c showcases plots of the true error ($\varepsilon_{t}$) acquired through the application of CDNN frameworks in the training of the experimental data against epochs. This figure demonstrates a reduction in the percentage of true error in both the initial and final trained cost functions, compared to values obtained using the previous DNN (2[2,2]HNS) framework. Specifically, the cost function decreases by 11.5% between the first two epochs when employing the formulated learning algorithms for this framework. Within the same range of epochs, significant reductions in true errors of 26.5%, 30.4%, 34.5%, and 36.5% were achieved using the formulated algorithms of CDNN frameworks characterized by 10, 8, 6, and 4 HNS, respectively. This reveals a gradual improvement in the modelling accuracy resulting from the reduction in the HNS (hyperparameter) within the frameworks.

Table 1 presents computed data from CDNN, displaying estimated values for synaptic weight ($w_{i}$), biases ($b_{k}$), epochs, true error ($\varepsilon_{t}$), approximate error ($\varepsilon_{a}$), overall cost function (C), average mass of degradation ($m_{ave}$), uncertainty (Δ), time (t), and selected learning rate (L). Figure 5c and Table 1 indicate that the CDNN (2[2,2]) framework (i.e., four hidden neurons) achieved the lowest true error (3.691) and accurately captured the mass loss profiles for all TGA experimental data. The estimated overall mass average value, along with the associated uncertainty for the predicted response, is 77.072 ± 1.652, whereas the measured data yield a value of 77.203 ± 1.708. This comparison underscores the accuracy of the trained model. The bias represents an offset in the modelling training, while the synaptic weight signifies the hierarchical contributions or sensitivity of input parameters to the activation or output response [39]. For instance, in the optimized CDNN (2[2,2]) framework and Table 1, the estimated values for synaptic weights $w_{1}$, $w_{2}$, $w_{3}$ and $w_{4}$ are −0.034, 7.750, 1.063, and 0.760, respectively. The highest estimated synaptic weight value ($w_{2}=7.750$) corresponds to the contribution from temperature to hidden neuron $a_{1}$, and a comparable contribution from this parameter is also observed for hidden neuron. Sensitivity follows in the order of degradation temperature, degradation time, composition, and heating rate. The application of artificial neural networks (ANN) to predict the mass loss profiles of polypropylene in [44] indicates that temperature is the most significant parameter in the pyrolysis process. The minimal contribution from the heating rate, as shown by the least contributory synaptic weight value ($w_{1}=-0.034$), offsets the characteristics of the mass loss profile obtained from co-pyrolysis operations, as depicted in Figure 1. Figure 5d displays plots comparing the CDNN predicted and measured sample weight loss against the reduced degradation temperature of the pure DS sample, using a constant heating rate of 10 °C.min^−1^. The accuracy of the model gradually improves throughout the training process. Furthermore, the experimental and CDNN predicted data closely align, with an estimated coefficient of determination ($R^{2}$) of 0.942.

**Figure 6:**
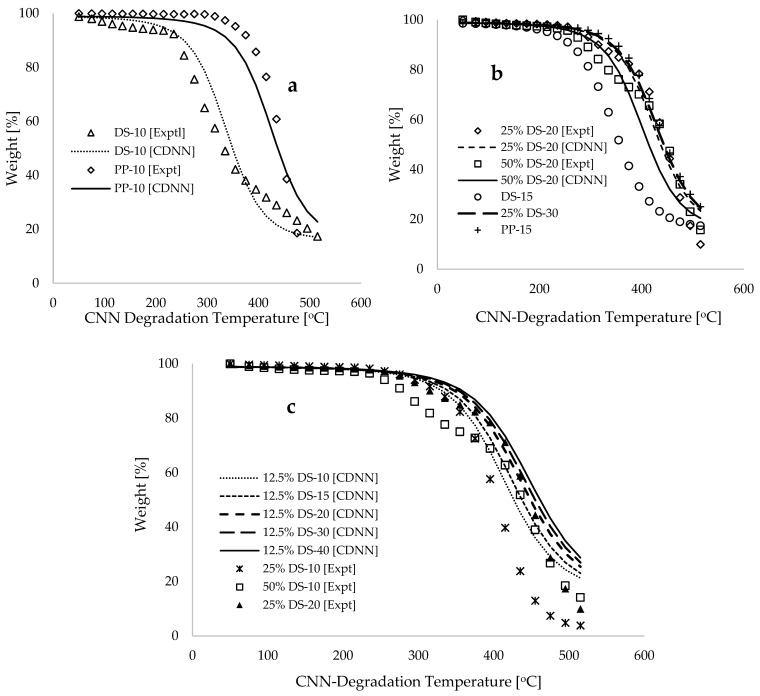
Plots illustrating the experimental and CDNN computed weight [%] against CNN degradation temperature [°C] for (**a**) DS and PP at 10 °C.min^−1^ of heating rate, and (**b**,**c**) DSP, DP and blends at obtained different heating rates.

Cross-validation of the trained model is essential for assessing its performance in a more generalized context. In Figure 6a, the experimental and CDNN predicted sample weight loss is plotted against degradation temperature for pure DS and PP materials, with a constant heating rate of 10 °C.min^−1^. Reasonable correlations between modelling and experiments are observed ($R^{2}$~0.942). Figure 6b displays the plots of experimental and CDNN projected data of sample weight loss against degradation for various heating rates and sample compositions. This demonstrates that, in blends with 25% and 50% DS sample compositions, the predicted mass loss profile at a heating rate of 20 °C.min^−1^ is consistent with the experimental data across most temperature ranges, with minor deviations that could be addressed through either extended modelling time or adjustment to modelling approach. Furthermore, Figure 6b illustrates that both experimental measurements and predictions for the blends are situated between the CDNN predictions for the individual pyrolysis of the DS and PP samples, even at a heating rate of 15 °C.min^−1^. This finding underscores the validity of the CDNN model. Figure 6c reveals that the projected predictions for a 12.5% DS composition result in a shift in the thermograms to higher temperature maxima as the heating rates increase from 10 to 40 °C.min^−1^. Importantly, all these projections at the 12.5% DS compositions fall within the experimental datasets for pure DS at 10 °C.min^−1^ and for pure PP materials. These trends are consistent with co-pyrolysis data of biomass-plastic, as substantiated in [12,16,17]. Furthermore, the results of this cross-validation indicate that increasing the addition of DS in the bio-composites consistently reduces their thermal stability, as evidenced by the shift in thermogram to lower temperatures and increases the ash content of the blends. It is important to note that during training, the reduction in the cost function occurs at a significantly slower rate as time and the number of epochs increase. For example, it required nearly four hours of training and 73,800 epochs to decrease the overall cost function from 1.404 (3.695% in true error) to 1.403 (3.691% in true error). This represents an insignificantly small difference given the substantial computational resources that have been utilized. Consequently, this highlights the limitations of the applicability of the CDNN model to such data, suggesting that the adoption of an alternative machine learning model may be considered for similar datasets in the future.

## 4. Kinetics and Thermodynamics of the Process

Obtaining the reaction pathway for this process is essential for understanding the impact of the addition of DS on the degradation of PP plastic and determining the energy requirements for the pyrolysis and co-pyrolysis processes. To achieve this, a thermo-kinetic assessment was conducted using both experimental and CDNN predicted data on sample weight loss. The assessment included pure DS, PP, and bio-composites (DS/PP). The Coats–Redfern (CR) [45] and the general Arrhenius [46] [ARH] model-fitting methods (see, Table 2) were employed to estimate the reaction pathway and kinetic parameters (activation energy [$E_{A}$] and pre-exponential factor [A]), which were then used to determine the thermodynamic parameters (change in entropy [∆S], enthalpy [∆H], and Gibbs free energy [∆G]) for the process (Table 2). The first-, second-, and third-order reactions (known as the F-Series [45,47]) were selected from the solid-state reaction mechanisms in [24] and used in conjunction with the chosen non-isothermal model-fitting methods (i.e., CR and ARH) to estimate the reaction mechanism. Furthermore, non-isothermal model-free methods, such as the Kissinger–Akahira–Sunose (KAS) and Flynn–Wall–Ozawa (FWO) methods [48,49,50,51,52], were utilized to estimate the thermo-kinetic parameters for the variable heating rates applied during for the TGA measurements.

Figure 1 illustrates that the presence of lignin in the DS biomass material inhibits degradation at elevated temperatures, while the PP plastic completely degrades to ash at temperatures below 500 °C. It is essential to emphasize that this study focuses on enhancing the thermal degradation of plastic by utilizing agricultural waste material. Consequently, the kinetics and thermodynamics of this process are anticipated to be assessed by analyzing the significant synergistic interactions arising from the decomposition of hemicellulose and cellulose present in the agricultural waste (DS biomass), as well as the complete decomposition of the PP plastic material. A prior study [14] conducted by this group provided thermograms for the thermal decomposition of date seed and high-density polyethylene blends, examining degradation temperatures ranging from 25 to 1000 °C and employing similar heating rates as those used in this study. The findings of this study [14] indicated that complete degradation of the DS biomass material was achieved at 650 °C during thermogravimetric analysis (TGA) measurements conducted at a heating rate of 10 °C·min^−1^. Furthermore, the study revealed that the thermogram of the DS biomass, conducted at 40 °C·min^−1^, plateaued for degradation temperatures between 600 and 1000 °C. This observation suggests that the application of the FWO and KAS model-free isoconvensional methods for estimating kinetic and thermodynamic parameters may overlook lower conversions at elevated temperatures for thermograms conducted at 40 °C·min^−1^. Vyazovkin et al. [53] concluded that a minimum of three heating rates is necessary for accurately estimating kinetic and thermodynamic parameters using model-free isoconvensional methods, whereas model-fitting methods require only a single heating rate. Furthermore, the DTG curves in Figure 1 clearly indicate that major peaks for co-pyrolysis of these materials occurred below 600 °C, suggesting that major chemical reactions occurred within this range. In view of these considerations, the kinetic and thermodynamic parameters for this study were estimated for conversions between 2.5 and 70 wt%, which were achieved at temperatures typically below 600 °C.

**Figure 7:**
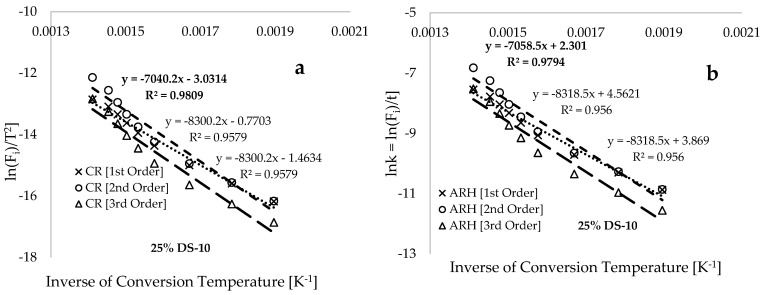

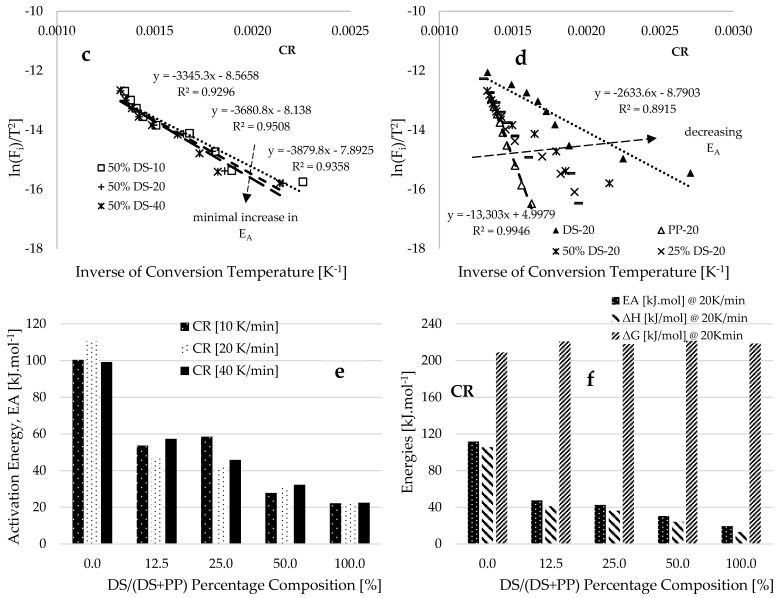
Plots of (**a**) Coats–Redfern’s $ln\left[\frac{\left(F_{i}\right)}{T^{2}}\right]$ at different reaction orders against inverse of conversion temperature [K^−1^], (**b**) Arrhenius’ $\ln\left[\frac{\left(F_{i}\right)}{T^{2}}\right]$ at different reaction orders against inverse of conversion temperature [K^−1^], (**c**) Coats–Redfern’s $ln\left[\frac{\left(F_{i}\right)}{T^{2}}\right]$ at different heating rates against inverse of conversion temperature [K^−1^], (**d**) Coats–Redfern’s $ln\left[\frac{\left(F_{i}\right)}{T^{2}}\right]$ at varied PD/PP compositions against inverse of conversion temperature [K^−1^], (**e**) activation energy, and (**f**) energies [kJ.mol^−1^] against percentage composition of DS in the composites.

Figure 7 illustrates plots of $ln(F_{i}/T^{2})$ versus the inverse of conversion temperature ($T^{-1}[K^{-1}])$ for conversion ranging between 2.5 and 70 wt% using the Coats–Redfern method (Figure 7a), Arrhenius method (Figure 7b), constant sample composition and varied heating rate (Figure 7c), and constant heating rate and varied sample composition (Figure 7d). As a general observation, plots in Figure 7a,b demonstrate that $ln(F_{i}/T^{2})$ is inversely linear with ($T^{-1}[K^{-1}])$ and the 1st order reaction yields the highest values of the coefficient of determination ($R^{2}$) compared to the other selected solid-state reaction mechanisms (SSRMs). A previous study by Zhou et al. [54] confirmed the suitability of the 1st order SSRM as the reaction mechanism for the co-pyrolysis of low volatile coal and polypropylene blends. Li et al. [52] and Baloch et al. [55] demonstrated that the application of the Coats–Redfern model-fitting methods resulted in the attainment of 1.0 and 0.741, respectively, as the order of the reaction for the thermal degradation of polypropylene. This 1st order SSRM was then used to estimate the thermo-kinetic parameters of the process, as presented in Table 3. Figure 7c shows that variation in the heating rate of the process for the 50% DS sample has minimal effect on the mass profile loss. However, Figure 7d,e illustrate that consistent increases in DS in the blends shift the non-linear plots to the temperature minima and consequently lead to a reduction in the activation and thermal stability of the bio-composites. Figure 7e,f serve as evidence that the predicted estimated values of the thermogram at 12.5% DS reflect the validity of the trained CDNN models, as these predicted values lie within the thermo-kinetic parameters estimated using experimental TGA data for the pure PP and 25% DS sample composition in the blends.

Table 3 demonstrates that the estimated activation energy values for pure PP at the different heating rates are roughly five times greater than those of pure DS, while the estimated values for the blends fall between the values for the individual materials. For instance, the estimated average activation energy values are as follows: 103.410 kJ.mol^−1^ for pure PP, 52.716 kJ.mol^−1^ for 12.5% DS, 48.834 kJ.mol^−1^ for 25% DS, 30.224 kJ.mol^−1^ for 50% DS, and 22.175 kJ.mol^−1^ for pure DS. These findings indicate that the activation energy ($E_{A})$ decreases as the quantity of DS in the bio-composite increases. Currently, there is limited or nonexistent information available on the $E_{A}$ values for pure DS and DS/PP blends. However, several studies have reported on the $E_{A}$ values for polypropylene. For example, Das and Tiwari [56] estimated the range of activation energy values for polypropylene to be 124–187 kJ.mol^−1^ using the isoconventional FWO, KAS, and STK model-free methods. Niser et al. [57] employed the FWO model-free kinetic method and obtained values ranging from 102 to 172.08 kJ.mol^−1^ for the TGA of PP conducted at 2, 12, 20, and 30 °C.min^−1^. Another study by [46] achieved average values of 106 kJ.mol^−1^ (FWO model), 108 kJ.mol^−1^ (KAS model), and 112 kJ.mol^−1^ (STK model) for PP plastic. Table 3 reveals that the estimated activation energy values for pure DS, PP, and DS/PP blends fall within the ranges of 18.783–22.439 kJ.mol^−1^, 99.228–111.748 kJ.mol^−1^, and 27.211–58.684 kJ.mol^−1^, respectively. The slight variations in values recorded in Table 3 are likely due to differences in heating rates [19], symmetric error between the two model-fitting methods [58], the equation origin [59], and the difference in non-linearity [60].

Table 3 shows that the estimated values for change in enthalpy (∆H) and Gibbs free energy (∆G) are positive throughout the process, confirming that the main reaction is endothermic and non-spontaneous, respectively, and would require heat to break down the materials. The estimated average values for ∆H for the pure PP, 12.5% DS, 25.0% DS, 50.0% DS, and pure DS are 98.367, 46.633, 43.061, 23.650, and 13.353 kJ·mol^−1^, respectively, suggesting that the enthalpy change for the process decreases with increasing addition of DS in the blends (see Figure 7f). Dobrzyńska-Mizera et al. [22] estimated the change in enthalpy for the thermal degradation of PP as 110.2 kJ·mol^−1^, and this value was reported to consistently decrease up to 70.2 kJ·mol^−1^ with increasing addition of walnut shells to the PP mixture. Table 3 shows that the entropy changes for this process are estimated to be all negative, indicating that the process is irreversible and non-equilibrium. However, the closeness of the entropy data to zero suggests that the process is less disordered [50,61,62].

**Figure 8:**
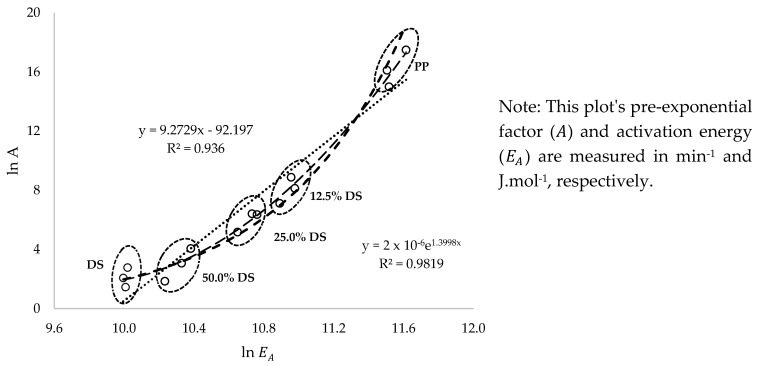
The kinetic compensation effect is illustrated by the plots of $ln\left(A\right)$ against $ln\left(E_{A}\right)$.

Estimated thermo-kinetic data presented in Table 3 indicate that the pre-exponential factors (A) obtained for the DS, PP, and DS/PP blends fall within the ranges of 4.31–16.226 min^−1^, 3.264 × 106–1.976 × 107 min^−1^, and 6.373–7.047 × 105 min^−1^, respectively. These findings suggest that changes in the activation energy ($E_{A}$) of the process influence the pre-exponential factors (A). Specifically, an increase in one leads to a corresponding increase in the other, and vice versa. According to Açıkalın and Üniversitesi [48], this phenomenon is referred to as the kinetic compensation effect, where alterations in the reaction rate of the process at a given time (*t*) and degradation temperature (*T*) impact both the $E_{A}$ and A. Janković et al. [49] established a linear mathematical relationship between the natural logarithm of these two parameters, expressed as $\ln A=\propto +\beta \cdot \ln E_{A}$ (where ∝ and β are constants), for the pyrolysis of swollen poly(acrylic acid) hydrogen. Figure 8 depicts a graph of ln⁡A against $\ln E_{A}$, estimated using Coats–Redfern model-fitting methods for all samples. The analysis reveals a coefficient of determination ($R^{2}$) of 0.936 for a linear fit, with ∝ and β estimated as −92.197 and 9.273, respectively. However, a more accurate correlation of determination ($R^{2}\sim 0.982$) is achieved when an exponential fit is applied to the data, indicating that the pre-exponential factor (A) increases exponentially with the rising value of $E_{A}$ for the process. This observation suggests that the TGA co-pyrolysis process of the biomass-plastic leads to rapid chemical reactions, resulting in an exponential growth in molecular collision, cleavage, disintegration, and rearrangement of molecules at higher temperatures [53,62,63,64]. Moreover, the non-linear exponential fit of the data (Figure 8) may be attributed to the effects imposed by the two-stage process of the bio-composites (i.e., the DS/PP blends).

**Figure 9:**
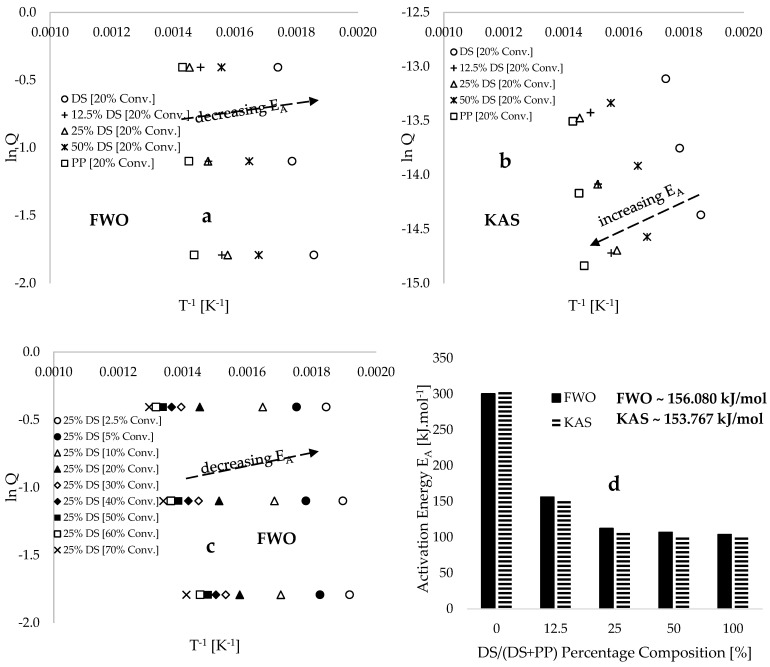
Plots of (**a**) FWO’s lnQ against the inverse of conversion temperature T^−1^ [K^−1^] for a 20% constant conversion and varied material compositions, (**b**) KAS’ $\ln\left[\frac{Q}{T^{2}}\right]$ against the inverse of conversion temperature T^−1^ [K^−1^] for a 20% constant conversion and varied material compositions, (**c**) FWO’s lnQ against the inverse of conversion temperature T^−1^ [K^−1^] for a 25% constant sample composition and varied conversions, and (**d**) overall activation energy [kJ.mol^−1^] against percentage composition of DS in the composites.

The Flynn–Wall–Ozawa (FWO) and Kissinger–Akahira–Sunose (KAS) model-free isoconvensional methods (refer to equations in Table 2) were employed to estimate the kinetic and thermodynamic parameters for this process. Figure 9a plots FWO’s lnQ against the inverse of the conversion temperature $T^{-1}[K^{-1}]$ at varying sample compositions, while Figure 9b illustrates analogous plots with kinetic data evaluated using the KAS model-free method. Figure 9c displays plots of FWO’s lnQ against the inverse of the conversion temperature $T^{-1}[K^{-1}]$ for a 25% DS sample composition at different conversions ranging from 2.5 to 70 wt%. A common feature of both Figure 9a,b are the consistent shifts in the linear inverse trends toward the temperature maxima with increasing plastic composition in the blend. This phenomenon indicates that the high thermal conductivity [33] and low moisture content [22] of the plastics play a significant role in slowing degradation at lower temperatures, particularly during the initiation stage of the degradation process. Consequently, an increase in activation energy was observed alongside a consistent increase in plastic composition, as demonstrated in Figure 9d and Table 4, utilizing both FWO and KAS model-free methods. Specifically, overall activation energy values ranging from 104.003 to 300.572 kJ.mol^−1^ and 100.103 to 305.851 kJ.mol^−1^ were obtained using the FWO and KAS models, respectively.

Figure 9c illustrates that the linear inverse trends at 2.5, 5.0, and 10.0 wt% exhibit analogous patterns in their characteristic slopes, indicating that moisture content in the samples is lost during this initial stage of degradation [24]. The pronounced gap between the linear inverse trends at 20 to 50 wt% conversions may be interpreted as a series of degradation steps resulting from the decomposition of hemicellulose and cellulose components in the biomass, as well as the degradation of the plastic present in the composite [22,53]. Beyond 60 wt%, the degradation can be attributed to the decomposition of lignin components within the biomass in the composites at elevated temperatures.

In comparison, the estimated overall average values of activation energies using the CR and ARH model-fitting methods are 51.471 and 51.221 kJ·mol^−1^, respectively, while the values of 156.080 and 153.767 kJ·mol^−1^ were estimated as the overall average values of activation energies using the FWO and KAS model-free methods, respectively. The deviation between the estimated values derived from the model-fitting and model-free methods could be attributed to the lower coefficient of determination obtained from fitting the kinetic data utilizing the model-fitting approaches, particularly at lower conversions and temperatures, as reported in [65]. Furthermore, the development of multi-step degradation resulting from the decomposition of hemicellulose and cellulose components in the biomass further decreases the linearity of the trend observed in the applicability of the model-fitting approaches. It is important to note that the hydrophilic nature of biomass materials results in low adhesion to plastic materials, which are predominantly hydrophobic [66,67]. Consequently, significant differences in particle size among the samples may lead to variations in the contact surfaces [68] between the materials. For instance, smaller particle sizes of DS biomass imply that a greater number of thermal contact surfaces are present within a given sample weight, potentially reducing the thermal stability of the composite. In this study, meticulous care was taken to ensure that both the plastic and biomass materials used are in powder form with similar particle sizes within the range of 100–200 µm to minimize the effects imposed by such operating parameters on the degradation of the materials. Therefore, a comprehensive experimental and modelling study is recommended for future research to investigate the effects of pore-structure-related parameters—including particle size, pore surface, packing density, tortuosity, and permeability—on the co-pyrolysis of randomly packed DS and PP materials. Additionally, further investigation should explore the application of various deep learning techniques in the formulation of algorithms and the determination of appropriate arbitrary constants for predicting experimentally measured data from differential thermogravimetric analysis (DTA) and differential scanning calorimetry (DSC) for these materials.

## 5. Conclusions

The present study evaluates the thermal decomposition (pyrolysis and co-pyrolysis) of date seed (DS), polypropylene homopolymer (PP), and their composites (DS/PP) using TGA data, machine learning convolutional deep neural networks (CDNN), and an analysis of the kinetics and thermodynamics. The decomposition temperature range was set between 25 and 600 °C, with heating rates of 10, 20, and 40 °C.min^−1^. Results indicate that DS exhibits a lower degradation temperature compared to PP, attributed to higher moisture content, cellulose, and hemicellulose in DS biomass. PP plastic degradation occurred via a single-step process, while incorporating DS into PP (i.e., blends) modified the intermediate stage, transforming it into a two-step process. The use of machine learning Convolutional Deep Neural Network (CDNN) on empirical data allowed for the filtering and estimation of crucial data points from each thermogram. This approach enables the development of algorithms that minimize the overall cost function and reduce the true error percentage (3.691%) between the model and experimental results. The CDNN framework in this study is optimized with four hidden neurons and 292,737 epochs, achieving a satisfactory correlation between the model and experiment. Additionally, cross-validation ensured that the model exhibits a generalized pattern within the experimental conditions. Sensitivity analysis of the CDNN synaptic weights and biases shows that the degradation process is significantly influenced by degradation temperature, followed by time, material composition, and heating rate.

The kinetics and thermodynamics of the process were investigated using the CR and ARH model-fitting approaches, alongside the FWO and KAS model-free isoconvensional methods. These models were employed to determine the activation energy ($E_{A}$), pre-exponential factor (*A*), change in enthalpy (∆H), entropy (∆S), and Gibbs free energy (∆G) for the process. The analysis revealed that the 1st order reaction mechanism showed the highest correlation of determination for all materials. The estimated overall activation energy values for the process, as determined by these methods, are 51.471, 51.221, 156.080, and 153.767 kJ.mol^−1^, respectively. Notably, the estimated values for the blends were within the range associated with the pyrolysis of individual materials. Furthermore, the kinetic compensation effect demonstrated an exponential increase ($R^{2}\sim 0.982$) in the pre-exponential factor (*A*) with increasing estimated values of $E_{A}$. The estimated values of ∆H and ∆G were positive throughout, indicating that the process is endothermic and non-spontaneous. In contrast, the estimated values of ∆S were close to zero, suggesting that the process is relatively less disordered.

## Figures and Tables

**Table 1:** CDNN computed data frameworks with 10, 8, 6, and 4 hidden neurons (HNS).

DNN Framework		$b_{1}$	$w_{1}$	$w_{2}$	$w_{3}$	$w_{4}$	$b_{2}$	$w_{5}$	$w_{6}$	$w_{7}$	$w_{8}$	$b_{3}$	$w_{9}$	
CDNN (2[5,5] HNS)		2.396	0.007	−5.813	−0.833	−0.565	2.396	0.007	−5.813	−0.833	−0.565	2.396	0.007	
CDNN (2[4,4] HNS)		4.075	0.018	−7.223	−1.016	−0.706	4.075	0.018	−7.223	−1.016	−0.706	4.075	0.018	
CDNN (2[3,3] HNS)		4.559	0.024	−7.814	−1.092	−0.765	4.559	0.024	−7.814	−1.092	−0.765	4.559	0.024	
CDNN (2[2,2] HNS)		−5.052	−0.034	7.750	1.065	0.760	−5.052	−0.034	7.750	1.065	0.760	0.000	0.000	
DNN (2[2,2] HNS)		4.438	−0.034	−7.477	−1.053	−0.658	4.438	−0.034	−7.477	−1.053	−0.658	0.000	0.000	
$w_{10}$	$w_{12}$	$w_{12}$	$b_{4}$	$w_{13}$	$w_{14}$	$w_{15}$	$w_{16}$	$b_{5}$	$w_{17}$	$w_{18}$	$w_{19}$	$w_{20}$	$b_{6}$	
−5.813	−0.833	−0.565	2.396	0.007	−5.813	−0.833	−0.565	2.396	0.007	−5.813	−0.833	−0.565	−4.235	
−7.223	−1.016	−0.706	4.075	0.018	−7.223	−1.016	−0.706	0.000	0.000	0.000	0.000	0.000	−6.546	
−7.814	−1.092	−0.765	0.000	0.000	0.000	0.000	0.000	0.000	0.000	0.000	0.000	0.000	−7.061	
0.000	0.000	0.000	0.000	0.000	0.000	0.000	0.000	0.000	0.000	0.000	0.000	0.000	1.069	
0.000	0.000	0.000	0.000	0.000	0.000	0.000	0.000	0.000	0.000	0.000	0.000	0.000	−6.374	
$w_{21}$	$w_{22}$	$w_{23}$	$w_{24}$	$w_{25}$	$b_{7}$	$w_{26}$	$w_{27}$	$w_{28}$	$w_{29}$	$w_{30}$	$b_{8}$	$w_{31}$	$w_{32}$	
1.801	1.801	1.801	1.801	1.801	−4.235	1.801	1.801	1.801	1.801	1.801	−4.235	1.801	1.801	
2.126	2.126	2.126	2.126	0.000	−6.546	2.126	2.126	2.126	2.126	0.000	−6.546	2.126	2.126	
2.833	2.833	2.833	0.000	0.000	−7.061	2.833	2.833	2.833	0.000	0.000	−7.061	2.833	2.833	
−5.838	−5.838	0.000	0.000	0.000	1.069	−5.838	−5.838	0.000	0.000	0.000	0.000	0.000	0.000	
3.975	3.975	0.000	0.000	0.000	−6.374	3.975	3.975	0.000	0.000	0.000	0.000	0.000	0.000	
$w_{33}$	$w_{34}$	$w_{35}$	$b_{9}$	$w_{36}$	$w_{37}$	$w_{38}$	$w_{38}$	$w_{40}$	$b_{10}$	$w_{41}$	$w_{42}$	$w_{43}$	$w_{44}$	
1.801	1.801	1.801	−4.235	1.801	1.801	1.801	1.801	1.801	−4.235	1.801	1.801	1.801	1.801	
2.126	2.126	0.000	−6.546	2.126	2.126	2.126	2.126	0.000	0.000	0.000	0.000	0.000	0.000	
2.833	0.000	0.000	0.000	0.000	0.000	0.000	0.000	0.000	0.000	0.000	0.000	0.000	0.000	
0.000	0.000	0.000	0.000	0.000	0.000	0.000	0.000	0.000	0.000	0.000	0.000	0.000	0.000	
0.000	0.000	0.000	0.000	0.000	0.000	0.000	0.000	0.000	0.000	0.000	0.000	0.000	0.000	
$w_{45}$	$b_{11}$	$w_{46}$	$w_{47}$	$w_{48}$	$w_{49}$	$w_{50}$	C	Epochs	${Ɛ}_{a}$	${Ɛ}_{t}$	$m_{ave}$	Δ	L	Time, t (mins)
1.801	−2.273	1.351	1.351	1.351	1.351	1.351	1.430	270,391	0.122	4.349	77.086	1.656	2.000	459.267
0.000	−1.830	1.850	1.850	1.850	1.850	0.000	1.413	338,771	0.065	4.094	77.084	1.654	2.000	572.483
0.000	−1.762	2.641	2.641	2.641	0.000	0.000	1.410	372,127	0.078	3.891	77.079	1.653	2.000	617.767
0.000	−1.627	4.223	4.223	0.000	0.000	0.000	1.403	292,737	0.035	3.691	77.072	1.652	1.000	497.800
0.000	−2.504	4.228	4.228	0.000	0.000	0.000	4.901	259,510.0	0.172	4.585	66.223	2.144	2.000	440.637

**Table 2:** Selected kinetic and thermodynamics equations for the analysis of TGA data.

Model-Fitting Methods for Single Heating Reaction Rate		
Coats–Redfern, (CR)	$\ln\left[\frac{\left(F_{i}\right)}{T^{2}}\right]=\ln\left(\frac{A\cdot R}{Q\cdot E_{A}}\right)-\left(\frac{E_{A}}{R}\right)\cdot \frac{1}{T}$	F-Series of the Solid-State Reaction Mechanisms (SSRM): [45]
Arrhenius (ARH)	$\ln\left[k\right]=\ln\left[\frac{\left(F_{i}\right)}{t}\right]=ln(A)-\left(\frac{E_{A}}{R}\right)\cdot \frac{1}{T}$	1st order reaction $\left(F_{1}\right)$ = [−$ln\left(1-x_{i}\right)$] 2nd order reaction $\left(F_{2}\right)={\left[(1-x_{i}\right)}^{-1}-1$] 3rd order reaction $\left(F_{3}\right)=$ $\left[{\left(1-x_{i}\right)}^{-1}-1\right]/2$ where $x_{i}=\frac{W_{o}-W_{t}}{W_{o}-W_{f}}\;is\;the\;conversion,$ $W_{o}$ is the initial weight, $W_{t}$ is the weight at time t, and $W_{f}$ is the final weight.
**Model-free methods for variable heating reaction rates**		
Flynn–Wall–Ozawa (FWO)	$ln[Q]=ln\left(\frac{A\cdot E_{A}}{R\cdot \left(g\left(x_{i}\right)\right)}\right)-5.331-1.052\left(\frac{E_{A}}{R}\right)\cdot \frac{1}{T}$	
Kissinger–Akahira–Sunose (KAS)	$ln[Q/T^{2}]=\ln\left(\frac{A\cdot R}{E_{A}\cdot \left(g\left(x_{i}\right)\right)}\right)-\left(\frac{E_{A}}{R}\right)\cdot \frac{1}{T}$	
**Thermodynamics parameters**		
Change in entropy [47]	$∆S=R\cdot \ln\left(\frac{A\cdot h}{K\cdot T_{M}}\right)$	$\mathrm{where}\;E_{A}$ is the activation energy (kJ.mol^−1^), R is the gas constant in J.mol^−1^.K^−1^, Q is the heating rate (°C.min^−1^), h denotes Planck’s constant (6.626 × 10^−34^ J.s^−1^), K is the Boltzmann constant (1.3806 × 10^−23^ J.K^−1^), and $T_{M}$ is the final or maximum temperature of constant conversion (K).
Change in enthalpy [47]	$∆H=E_{A}-R\cdot T_{M}$	
Change in Gibbs free energy [46]	$∆G=∆H-T_{M}\cdot ∆S$	

**Table 3:** Estimated kinetic and thermodynamic parameters using the CR and ARH model-fitting methods for single heating rate.

Sample	Model	Q	$T_{m}$	n	$R^{2}$ [-]	$E_{A}$	A	∆S	∆H	∆G
PP-10	CR	10	735.861	1st	0.994	100.392	3.264 × 10^6^	−0.162	94.274	213.309
	ARH	10			0.994	101.464	8.075 × 10^8^	−0.116	95.346	180.667
DS-10	CR	10	700.785		0.928	22.189	4.310	−0.274	16.363	208.315
	ARH	10			0.869	18.783	365.531	−0.237	12.957	179.037
**12.5% DS-10**	CR	10	744.979		0.976	53.698	1.229 × 10^3^	−0.227	47.505	216.927
	ARH	10			0.973	53.795	2.537 × 10^5^	−0.183	47.601	184.010
25% DS-10	CR	10	708.551		0.981	58.532	3.397 × 10^3^	−0.219	52.641	207.495
	ARH	10			0.979	58.684	7.047 × 10^5^	−0.174	52.793	176.219
50% DS-10	CR	10	742.459		0.930	27.813	6.373	−0.271	21.640	222.949
	ARH	10			0.915	27.211	1.136 × 10^3^	−0.228	21.038	190.353
PP-20	CR	20	739.685		0.995	110.601	3.940 × 10^7^	−0.141	104.451	208.820
	ARH	20			0.994	111.748	1.976 × 10^10^	−0.089	105.599	171.731
DS-20	CR	20	753.302		0.892	21.896	8.017	−0.269	15.633	218.535
	ARH	20			0.823	19.283	1.705 × 10^3^	−0.225	13.020	182.356
**12.5% DS-20**	CR	20	769.504		0.937	47.152	575.031	−0.234	40.754	220.820
	ARH	20			0.934	47.398	2.464 × 10^5^	−0.184	41.000	182.295
25% DS-20	CR	20	746.332		0.963	42.163	176.106	−0.244	35.958	217.754
	ARH	20			0.962	42.503	2.464 × 10^5^	−0.193	36.298	180.385
50% DS-20	CR	20	754.562		0.951	30.602	21.512	−0.261	24.329	221.389
	ARH	20			0.943	30.393	8.390 × 10^3^	−0.212	24.120	183.752
PP-40	CR	40	751.106		0.978	99.228	9.950 × 10^6^	−0.153	92.983	207.653
	ARH	40			0.978	100.400	1.004 × 10^10^	−0.095	94.155	165.634
DS-40	CR	40	744.401		0.858	22.439	16.226	−0.263	16.251	212.318
	ARH	40			0.796	20.270	7.742 × 10^3^	−0.212	14.081	171.977
**12.5% DS-40**	CR	40	783.562		0.956	57.300	7.173 × 10^3^	−0.213	50.786	217.819
	ARH	40			0.953	57.811	6.471 × 10^6^	−0.157	51.297	174.001
25% DS-40	CR	40	771.670		0.946	45.807	607.424	−0.234	39.391	219.630
	ARH	40			0.946	46.507	5.706 × 10^5^	−0.177	40.091	176.414
50% DS-40	CR	40	754.976		0.936	32.257	57.970	−0.253	25.980	216.929
	ARH	40			0.926	32.069	4.540 × 10^4^	−0.177	25.792	174.916

NB: Q is the heating rate [K.min^−1^], $T_{m}$ is the maximum temperature of constant conversion [K], n is the reaction order [-], $R^{2}$ is the coefficient of determination [-], $E_{A}$ is the activation energy [kJ.mol^−1^], A is the pre-exponential factor [min^−1^], ∆S is the change in entropy [kJ.mol^−1^.K^−1^], ∆H is the change in enthalpy [kJ.mol^−1^], and ∆G is the change in Gibbs free energy [kJ.mol^−1^].

**Table 4:** Estimated kinetic and thermodynamic parameters using the FWO and KAS model-free methods for variable heating rates.

Sample	Model	R^2^ [-]	E_A_ [kJ.mol^−1^]	A [s^−1^]	ΔS [kJ.mol^−1^.K^−1^]	ΔH [kJ.mol^−1^]	ΔG [kJ.mol^−1^]
DS	FWO	0.987	104.053	4.488 × 10^1^	−0.111	99.222	160.985
	KAS	0.984	100.103	2.724 × 10^1^	−0.121	95.272	162.926
12.5% DS	FWO	**0.978**	**156.257**	**2.680 × 10^11^**	**−0.056**	**150.627**	**189.767**
	KAS	**0.973**	**153.388**	**2.243 × 10^11^**	**−0.061**	**147.757**	**190.662**
25% DS	FWO	0.974	112.490	2.392 × 10^13^	−0.117	106.790	192.520
	KAS	0.969	107.368	2.276 × 10^13^	−0.145	101.667	205.122
50% DS	FWO	0.9447	107.0284	1.424 × 10^11^	−0.1178	101.6268	179.2595
	KAS	0.9347	102.1279	2.751 × 10^8^	−0.1439	96.7263	190.3027
PP	FWO	0.984	300.572	4.176 × 10^22^	0.142	294.734	193.314
	KAS	0.981	305.851	1.589 × 10^23^	0.161	300.012	186.267

## Data Availability

Data will be made available upon request.

## Supplementary Materials

The following supporting information can be downloaded at: https://www.mdpi.com/article/10.3390/polym17030307/s1.
