# Supplementary material for: Thermal Decomposition of Date Seed/Polypropylene Homopolymer: Machine Learning CDNN, Kinetics, and Thermodynamics
Source: Polymers (Basel). 2025 Jan 23;17(3):307. doi: 10.3390/polym17030307 (PMC11820839; doi:10.3390/polym17030307)
Supplement: Supplementary file 1 [file polymers-17-00307-s001.zip › polymers-3382766-supplementary.pdf]

Thermal Decomposition of Date Seed/Polypropylene Homopolymer: Machine Learning CDNN, Kinetics, and Thermodynamics

Zaid Abdulhamid Alhulaybi & Abdulrazak Jinadu Otaru

Department of Chemical Engineering, College of Engineering, King Faisal University, Al Ahsa, Saudi Arabia.
Correspondence: zalhulaybi@kfu.edu.sa and aotaru@kfu.edu.sa
A.J Otaru's ORCID No: 0000-0002-3057-4991

Formulation of the Learning Algorithms for the DNN Framework (Fig 2)

Applying the backpropagation method for the determination of sum weight (z_i), Sigma activation function (a_i)and overall cost function (C_i) using Eqns 1-3 in the main article, the following are achieved for DNN framework in Fig 2.

$$z_{11} = b_{11} + w_{46}.a_6 + w_{47}.a_7 + w_{48}.a_8 + w_{49}.a_9 + w_{50}.a_{10}$$ Eqn 4.0

$$a_{11} = \sigma'(z_{11}) = \frac{1}{(1+e^{-z_{11}})}$$ Eqn 4.1

$$C_i = (y - a_{11})^2$$ Eqn 4.2

where $a_6 = \sigma'(z_6) = \frac{1}{(1+e^{-z_6})}$ & $z_6 = b_6 + w_{21}.a_1 + w_{22}.a_2 + w_{23}.a_3 + w_{24}.a_4 + w_{25}.a_5$ Eqn 4.3

$$a_7 = \sigma'(z_7) = \frac{1}{(1+e^{-z_7})} \; \& \; z_7 = b_7 + w_{26}.a_1 + w_{27}.a_2 + w_{28}.a_3 + w_{29}.a_4 + w_{30}.a_5$$ Eqn 4.4

$$a_8 = \sigma'(z_8) = \frac{1}{(1+e^{-z_8})} \; \& \; z_8 = b_8 + w_{31}.a_1 + w_{32}.a_2 + w_{33}.a_3 + w_{34}.a_4 + w_{35}.a_5$$ Eqn 4.5

$$a_9 = \sigma'(z_9) = \frac{1}{(1+e^{-z_9})} \; \& \; z_9 = b_9 + w_{36}.a_1 + w_{37}.a_2 + w_{38}.a_3 + w_{39}.a_4 + w_{40}.a_5$$ Eqn 4.6

$$a_{10} = \sigma'(z_{10}) = \frac{1}{(1+e^{-z_{10}})} \; \& \; z_{10} = b_{10} + w_{41}.a_1 + w_{42}.a_2 + w_{43}.a_3 + w_{44}.a_4 + w_{44}.a_5$$ Eqn 4.7

$$a_1 = \sigma'(z_1) = \frac{1}{(1+e^{-z_1})} \; \& \; z_1 = b_1 + w_1.x_1 + w_2.x_2 + w_3.x_3 + w_4.x_4$$ Eqn 4.8

$$a_2 = \sigma'(z_2) = \frac{1}{(1+e^{-z_2})} \; \& \; z_2 = b_2 + w_5.x_1 + w_6.x_2 + w_7.x_3 + w_8.x_4$$ Eqn 4.9

$$a_3 = \sigma'(z_3) = \frac{1}{(1+e^{-z_3})} \; \& \; z_3 = b_3 + w_9.x_1 + w_{10}.x_2 + w_{11}.x_3 + w_{12}.x_4$$ Eqn 4.10

$$a_4 = \sigma'(z_4) = \frac{1}{(1+e^{-z_4})} \; \& \; z_4 = b_4 + w_{13}.x_1 + w_{14}.x_2 + w_{15}.x_3 + w_{16}.x_4$$ Eqn 4.11

$$a_5 = \sigma'(z_5) = \frac{1}{(1+e^{-z_5})} \; \& \; z_4 = b_5 + w_{17}.x_1 + w_{18}.x_2 + w_{19}.x_3 + w_{20}.x_4$$ Eqn 4.12

Cost optimisation function

The following steps are followed for the formulation of the learning algorithms for the cost optimisation functions.

1. **The overall cost function on output activation function 11**

The estimation of the cost function at this stage is dependent on the knowledge of the changes in bias (Δb) and weights (Δw) and these can be formulated as a test function of the input activation functions ($a_6 - a_{10}$), learning rate (L), sum weights and real output data.

$$w = -L.\frac{\partial C}{\partial w} \text{ and } \Delta b = -L.\frac{\partial C}{\partial b} \quad \text{Eqn 5.0}$$

where $$\frac{\partial C}{\partial b} = \left(\frac{\partial C}{\partial a}\right)\left(\frac{\partial a}{\partial z}\right)\left(\frac{\partial z}{\partial b}\right) \,\&\, \frac{\partial C}{\partial w} = \left(\frac{\partial C}{\partial a}\right)\left(\frac{\partial a}{\partial z}\right)\left(\frac{\partial z}{\partial w}\right) \quad \text{Eqn 5.1}$$

$$\left(\frac{\partial C}{\partial a_{11}}\right) = 2(a_{11} - y); \left(\frac{\partial a_{11}}{\partial z_{11}}\right) = \sigma'(z_{11}) = \frac{e^{z_{11}}}{(1+e^{z_{11}})^2}; \left(\frac{\partial z_{11}}{\partial w_{46}}\right) = a_6, \left(\frac{\partial z_{11}}{\partial w_{47}}\right) = a_7,$$

$$\left(\frac{\partial z_{11}}{\partial w_{48}}\right) = a_8, \left(\frac{\partial z_{11}}{\partial w_{49}}\right) = a_9, \left(\frac{\partial z_{11}}{\partial w_{50}}\right) = a_{10} \,\&\, \left(\frac{\partial z_{11}}{\partial b_{11}}\right) = 1 \quad \text{Eqn 5.2}$$

$$\left(\frac{\partial C}{\partial b_{11}}\right) = \left(\frac{\partial C}{\partial a_{11}}\right)\left(\frac{\partial a_{11}}{\partial z_{11}}\right)\left(\frac{\partial z_{11}}{\partial b_{11}}\right) \quad \text{Eqn 5.3a}$$

$$\left(\frac{\partial C}{\partial w_{46}}\right) = \left(\frac{\partial C}{\partial a_{11}}\right)\left(\frac{\partial a_{11}}{\partial z_{11}}\right)\left(\frac{\partial z_{11}}{\partial w_{46}}\right) \quad \text{Eqn 5.3b}$$

$$\left(\frac{\partial C}{\partial w_{47}}\right) = \left(\frac{\partial C}{\partial a_{11}}\right)\left(\frac{\partial a_{11}}{\partial z_{11}}\right)\left(\frac{\partial z_{11}}{\partial w_{47}}\right) \quad \text{Eqn 5.3c}$$

$$\left(\frac{\partial C}{\partial w_{48}}\right) = \left(\frac{\partial C}{\partial a_{11}}\right)\left(\frac{\partial a_{11}}{\partial z_{11}}\right)\left(\frac{\partial z_{11}}{\partial w_{48}}\right) \quad \text{Eqn 5.3d}$$

$$\left(\frac{\partial C}{\partial w_{49}}\right) = \left(\frac{\partial C}{\partial a_{11}}\right)\left(\frac{\partial a_{11}}{\partial z_{11}}\right)\left(\frac{\partial z_{11}}{\partial w_{49}}\right) \quad \text{Eqn 5.3e}$$

$$\left(\frac{\partial C}{\partial w_{50}}\right) = \left(\frac{\partial C}{\partial a_{11}}\right)\left(\frac{\partial a_{11}}{\partial z_{11}}\right)\left(\frac{\partial z_{11}}{\partial w_{50}}\right) \quad \text{Eqn 5.3f}$$

Hence: $$\Delta b_{11} = -L\left(\sum_{i=1}^{n}\left(\frac{\partial C}{\partial b_7}\right)\right)/n \quad \& \quad b_7 = b_7 + \Delta b_5 \quad \text{Eqn 5.4a}$$

$$\Delta w_{46} = -L\left(\sum_{i=1}^{n}\left(\frac{\partial C}{\partial w_{46}}\right)\right)/n \quad \& \; w_{46(New)} = w_{46(Old)} + \Delta w_{46} \quad \text{Eqn 5.4b}$$

$$\Delta w_{47} = -L\left(\sum_{i=1}^{n}\left(\frac{\partial C}{\partial w_{47}}\right)\right)/n \quad \& \; w_{47(New)} = w_{47(Old)} + \Delta w_{47} \quad \text{Eqn 5.4c}$$

$$\Delta w_{48} = -L\left(\sum_{i=1}^{n}\left(\frac{\partial C}{\partial w_{48}}\right)\right)/n \quad \& \; w_{48(New)} = w_{48(Old)} + \Delta w_{48} \quad \text{Eqn 5.4d}$$

$$\Delta w_{49} = -L\left(\sum_{i=1}^{n}\left(\frac{\partial C}{\partial w_{49}}\right)\right)/n \quad \& \; w_{49(New)} = w_{49(Old)} + \Delta w_{49} \quad \text{Eqn 5.4e}$$

$$\Delta w_{50} = -L\left(\sum_{i=1}^{n}\left(\frac{\partial C}{\partial w_{50}}\right)\right)/n \quad \& \; w_{50(New)} = w_{50(Old)} + \Delta w_{50} \quad \text{Eqn 5.4f}$$

2. The overall cost function on hidden neurons (HNs) $a_6 - a_{10}$

The changes in bias and weights at this stage is done as a test function of activation with respect to sum weight and sum weight with respect to individual weights and biases.

$$\frac{\partial C}{\partial b} = \left(\frac{\partial C}{\partial a}\right)\left(\frac{\partial a}{\partial z}\right)\left(\frac{\partial z}{\partial b}\right) \; \& \; \frac{\partial C}{\partial w} = \left(\frac{\partial C}{\partial a}\right)\left(\frac{\partial a}{\partial z}\right)\left(\frac{\partial z}{\partial w}\right) \qquad \text{Eqn 5.5}$$

where $\left(\frac{\partial z_{11}}{\partial a_6}\right) = w_{46}; \left(\frac{\partial z_{11}}{\partial a_7}\right) = w_{47}; \left(\frac{\partial z_{11}}{\partial a_8}\right) = w_{48}; \left(\frac{\partial z_{11}}{\partial a_9}\right) = w_{49}; \left(\frac{\partial z_{11}}{\partial a_{10}}\right) = w_{50}$ Eqn 5.6

$$\left(\frac{\partial C}{\partial a_6}\right) = \left(\frac{\partial C}{\partial a_{11}}\right)\left(\frac{\partial a_{11}}{\partial z_{11}}\right)\left(\frac{\partial z_{11}}{\partial a_6}\right) \quad \text{where } \left(\frac{\partial a_{11}}{\partial z_{11}}\right) = \sigma'(z_{11}) = \frac{e^{z_{11}}}{(1+e^{z_{11}})^2} \qquad \text{Eqn 5.7a}$$

$$\left(\frac{\partial C}{\partial a_7}\right) = \left(\frac{\partial C}{\partial a_{11}}\right)\left(\frac{\partial a_{11}}{\partial z_{11}}\right)\left(\frac{\partial z_{11}}{\partial a_7}\right) \qquad \text{Eqn 5.7b}$$

$$\left(\frac{\partial C}{\partial a_8}\right) = \left(\frac{\partial C}{\partial a_{11}}\right)\left(\frac{\partial a_{11}}{\partial z_{11}}\right)\left(\frac{\partial z_{11}}{\partial a_8}\right) \qquad \text{Eqn 5.7c}$$

$$\left(\frac{\partial C}{\partial a_9}\right) = \left(\frac{\partial C}{\partial a_{11}}\right)\left(\frac{\partial a_{11}}{\partial z_{11}}\right)\left(\frac{\partial z_{11}}{\partial a_9}\right) \qquad \text{Eqn 5.7d}$$

$$\left(\frac{\partial C}{\partial a_{10}}\right) = \left(\frac{\partial C}{\partial a_{11}}\right)\left(\frac{\partial a_{11}}{\partial z_{11}}\right)\left(\frac{\partial z_{11}}{\partial a_{10}}\right) \qquad \text{Eqn 5.7e}$$

$$\left(\frac{\partial C}{\partial b_6}\right) = \left(\frac{\partial C}{\partial a_6}\right)\left(\frac{\partial a_6}{\partial z_6}\right)\left(\frac{\partial z_6}{\partial b_6}\right) \quad \text{where } \left(\frac{\partial z_6}{\partial b_6}\right) = 1 \qquad \text{Eqn 5.8a}$$

$$\left(\frac{\partial C}{\partial w_{21}}\right) = \left(\frac{\partial C}{\partial a_6}\right)\left(\frac{\partial a_6}{\partial z_6}\right)\left(\frac{\partial z_6}{\partial w_{21}}\right) \quad \text{where } \left(\frac{\partial z_6}{\partial w_{21}}\right) = a_1 \; \& \left(\frac{\partial a_6}{\partial z_6}\right) = \sigma'(z_6) = \frac{e^{z_6}}{(1+e^{z_6})^2} \qquad \text{Eqn 5.8b}$$

$$\left(\frac{\partial C}{\partial w_{22}}\right) = \left(\frac{\partial C}{\partial a_6}\right)\left(\frac{\partial a_6}{\partial z_6}\right)\left(\frac{\partial z_6}{\partial w_{22}}\right) \quad \text{where } \left(\frac{\partial z_6}{\partial w_{22}}\right) = a_2 \qquad \text{Eqn 5.8c}$$

$$\left(\frac{\partial C}{\partial w_{23}}\right) = \left(\frac{\partial C}{\partial a_6}\right)\left(\frac{\partial a_6}{\partial z_6}\right)\left(\frac{\partial z_6}{\partial w_{23}}\right) \quad \text{where } \left(\frac{\partial z_4}{\partial w_{23}}\right) = a_3 \qquad \text{Eqn 5.8d}$$

$$\left(\frac{\partial C}{\partial w_{24}}\right) = \left(\frac{\partial C}{\partial a_6}\right)\left(\frac{\partial a_6}{\partial z_6}\right)\left(\frac{\partial z_6}{\partial w_{24}}\right) \quad \text{where } \left(\frac{\partial z_6}{\partial w_{24}}\right) = a_4 \qquad \text{Eqn 5.8e}$$

$$\left(\frac{\partial C}{\partial w_{25}}\right) = \left(\frac{\partial C}{\partial a_6}\right)\left(\frac{\partial a_6}{\partial z_6}\right)\left(\frac{\partial z_6}{\partial w_{25}}\right) \quad \text{where } \left(\frac{\partial z_6}{\partial w_{25}}\right) = a_5 \qquad \text{Eqn 5.8f}$$

Hence: $\Delta b_6 = -L\left(\sum_{i=1}^{n}\left(\frac{\partial C}{\partial b_6}\right)\right)/n \;\; \& \;\; b_{6(New)} = b_{6(Old)} + \Delta b_6$ Eqn 5.9a

$$\Delta w_{21} = -L\left(\sum_{i=1}^{n}\left(\frac{\partial C}{\partial w_{21}}\right)\right)/n \;\; \& \; w_{21(New)} = w_{21(Old)} + \Delta w_{21} \qquad \text{Eqn 5.9b}$$

$$\Delta w_{22} = -L\left(\sum_{i=1}^{n}\left(\frac{\partial C}{\partial w_{22}}\right)\right)/n \;\; \& \; w_{22(New)} = w_{22(Old)} + \Delta w_{22} \qquad \text{Eqn 5.9c}$$

$$\Delta w_{23} = -L\left(\sum_{i=1}^{n}\left(\frac{\partial C}{\partial w_{23}}\right)\right)/n \;\; \& \; w_{23(New)} = w_{23(Old)} + \Delta w_{23} \qquad \text{Eqn 5.9d}$$

$$\Delta w_{24} = -L\left(\sum_{i=1}^{n}\left(\frac{\partial C}{\partial w_{24}}\right)\right)/n \;\; \& \; w_{24(New)} = w_{24(Old)} + \Delta w_{24} \qquad \text{Eqn 5.9e}$$

$$\Delta w_{25} = -L\left(\sum_{i=1}^{n}\left(\frac{\partial C}{\partial w_{25}}\right)\right)/n \quad \& \; w_{25(New)} = w_{25(Old)} + \Delta w_{25}$$ Eqn 5.9f

$$\left(\frac{\partial C}{\partial b_7}\right) = \left(\frac{\partial C}{\partial a_7}\right)\left(\frac{\partial a_7}{\partial z_7}\right)\left(\frac{\partial z_7}{\partial b_7}\right)$$ where $\left(\frac{\partial z_7}{\partial b_7}\right) = 1$ Eqn 6.0a

$$\left(\frac{\partial C}{\partial w_{26}}\right) = \left(\frac{\partial C}{\partial a_7}\right)\left(\frac{\partial a_7}{\partial z_7}\right)\left(\frac{\partial z_7}{\partial w_{26}}\right)$$ where $\left(\frac{\partial z_7}{\partial w_{26}}\right) = a_1$ & $\left(\frac{\partial a_7}{\partial z_7}\right) = \sigma'(z_7) = \frac{e^{z_7}}{(1+e^{z_7})^2}$ Eqn 6.0b

$$\left(\frac{\partial C}{\partial w_{27}}\right) = \left(\frac{\partial C}{\partial a_7}\right)\left(\frac{\partial a_7}{\partial z_7}\right)\left(\frac{\partial z_7}{\partial w_{27}}\right)$$ where $\left(\frac{\partial z_7}{\partial w_{27}}\right) = a_2$ Eqn 6.0c

$$\left(\frac{\partial C}{\partial w_{28}}\right) = \left(\frac{\partial C}{\partial a_7}\right)\left(\frac{\partial a_7}{\partial z_7}\right)\left(\frac{\partial z_7}{\partial w_{28}}\right)$$ where $\left(\frac{\partial z_7}{\partial w_{28}}\right) = a_3$ Eqn 6.0d

$$\left(\frac{\partial C}{\partial w_{29}}\right) = \left(\frac{\partial C}{\partial a_7}\right)\left(\frac{\partial a_7}{\partial z_7}\right)\left(\frac{\partial z_7}{\partial w_{29}}\right)$$ where $\left(\frac{\partial z_7}{\partial w_{29}}\right) = a_4$ Eqn 6.0e

$$\left(\frac{\partial C}{\partial w_{30}}\right) = \left(\frac{\partial C}{\partial a_7}\right)\left(\frac{\partial a_7}{\partial z_7}\right)\left(\frac{\partial z_7}{\partial w_{30}}\right)$$ where $\left(\frac{\partial z_7}{\partial w_{30}}\right) = a_5$ Eqn 6.0f

Hence: $\Delta b_7 = -L\left(\sum_{i=1}^{n}\left(\frac{\partial C}{\partial b_7}\right)\right)/n \quad \& \; b_{7(New)} = b_{7(Old)} + \Delta b_7$ Eqn 6.1a

$$\Delta w_{26} = -L\left(\sum_{i=1}^{n}\left(\frac{\partial C}{\partial w_{26}}\right)\right)/n \quad \& \; w_{26(New)} = w_{26(Old)} + \Delta w_{26}$$ Eqn 6.1b

$$\Delta w_{27} = -L\left(\sum_{i=1}^{n}\left(\frac{\partial C}{\partial w_{27}}\right)\right)/n \quad \& \; w_{27(New)} = w_{27(Old)} + \Delta w_{27}$$ Eqn 6.1c

$$\Delta w_{28} = -L\left(\sum_{i=1}^{n}\left(\frac{\partial C}{\partial w_{28}}\right)\right)/n \quad \& \; w_{28(New)} = w_{28(Old)} + \Delta w_{28}$$ Eqn 6.1d

$$\Delta w_{29} = -L\left(\sum_{i=1}^{n}\left(\frac{\partial C}{\partial w_{29}}\right)\right)/n \quad \& \; w_{29(New)} = w_{29(Old)} + \Delta w_{29}$$ Eqn 6.1e

$$\Delta w_{30} = -L\left(\sum_{i=1}^{n}\left(\frac{\partial C}{\partial w_{30}}\right)\right)/n \quad \& \; w_{30(New)} = w_{30(Old)} + \Delta w_{30}$$ Eqn 6.1f

$$\left(\frac{\partial C}{\partial b_8}\right) = \left(\frac{\partial C}{\partial a_8}\right)\left(\frac{\partial a_8}{\partial z_8}\right)\left(\frac{\partial z_8}{\partial b_8}\right)$$ where $\left(\frac{\partial z_8}{\partial b_8}\right) = 1$ Eqn 6.2a

$$\left(\frac{\partial C}{\partial w_{31}}\right) = \left(\frac{\partial C}{\partial a_8}\right)\left(\frac{\partial a_8}{\partial z_8}\right)\left(\frac{\partial z_8}{\partial w_{31}}\right)$$ where $\left(\frac{\partial z_8}{\partial w_{31}}\right) = a_1$ & $\left(\frac{\partial a_8}{\partial z_8}\right) = \sigma'(z_8) = \frac{e^{z_8}}{(1+e^{z_8})^2}$ Eqn 6.2b

$$\left(\frac{\partial C}{\partial w_{32}}\right) = \left(\frac{\partial C}{\partial a_8}\right)\left(\frac{\partial a_8}{\partial z_8}\right)\left(\frac{\partial z_8}{\partial w_{32}}\right)$$ where $\left(\frac{\partial z_8}{\partial w_{32}}\right) = a_2$ Eqn 6.2c

$$\left(\frac{\partial C}{\partial w_{33}}\right) = \left(\frac{\partial C}{\partial a_8}\right)\left(\frac{\partial a_8}{\partial z_8}\right)\left(\frac{\partial z_8}{\partial w_{33}}\right)$$ where $\left(\frac{\partial z_8}{\partial w_{33}}\right) = a_3$ Eqn 6.2d

$$\left(\frac{\partial C}{\partial w_{34}}\right) = \left(\frac{\partial C}{\partial a_8}\right)\left(\frac{\partial a_8}{\partial z_8}\right)\left(\frac{\partial z_8}{\partial w_{34}}\right)$$ where $\left(\frac{\partial z_8}{\partial w_{34}}\right) = a_4$ Eqn 6.2e

$$\left(\frac{\partial C}{\partial w_{35}}\right) = \left(\frac{\partial C}{\partial a_8}\right)\left(\frac{\partial a_8}{\partial z_8}\right)\left(\frac{\partial z_8}{\partial w_{35}}\right)$$ where $\left(\frac{\partial z_8}{\partial w_{35}}\right) = a_5$ Eqn 6.2f

Hence: $\Delta b_8 = -L\left(\sum_{i=1}^{n}\left(\frac{\partial C}{\partial b_8}\right)\right)/n \quad \& \; b_{8(New)} = b_{8(Old)} + \Delta b_8$ Eqn 6.3a

$$\Delta w_{31} = -L\left(\sum_{i=1}^{n}\left(\frac{\partial C}{\partial w_{31}}\right)\right)/n \quad \& \; w_{31(New)} = w_{31(Old)} + \Delta w_{31}$$ Eqn 6.3b

$$\Delta w_{32} = -L\left(\sum_{i=1}^{n}\left(\frac{\partial C}{\partial w_{32}}\right)\right)/n \quad \& \; w_{32(New)} = w_{32(Old)} + \Delta w_{32}$$ Eqn 6.3c

$$\Delta w_{33} = -L\left(\sum_{i=1}^{n}\left(\frac{\partial C}{\partial w_{33}}\right)\right)/n \quad \& \; w_{33(New)} = w_{33(Old)} + \Delta w_{33}$$ Eqn 6.3d

$$\Delta w_{34} = -L\left(\sum_{i=1}^{n}\left(\frac{\partial C}{\partial w_{34}}\right)\right)/n \quad \& \; w_{34(New)} = w_{34(Old)} + \Delta w_{34}$$ Eqn 6.3e

$$\Delta w_{35} = -L\left(\sum_{i=1}^{n}\left(\frac{\partial C}{\partial w_{35}}\right)\right)/n \quad \& \; w_{35(New)} = w_{35(Old)} + \Delta w_{35}$$ Eqn 6.3f

$$\left(\frac{\partial C}{\partial b_9}\right) = \left(\frac{\partial C}{\partial a_9}\right)\left(\frac{\partial a_9}{\partial z_9}\right)\left(\frac{\partial z_9}{\partial b_9}\right) \quad \text{where } \left(\frac{\partial z_9}{\partial b_9}\right) = 1$$ Eqn 6.4a

$$\left(\frac{\partial C}{\partial w_{36}}\right) = \left(\frac{\partial C}{\partial a_9}\right)\left(\frac{\partial a_9}{\partial z_9}\right)\left(\frac{\partial z_9}{\partial w_{36}}\right) \quad \text{where } \left(\frac{\partial z_9}{\partial w_{36}}\right) = a_1 \; \& \; \left(\frac{\partial a_9}{\partial z_9}\right) = \sigma'(z_9) = \frac{e^{z_9}}{(1+e^{z_9})^2}$$ Eqn 6.4b

$$\left(\frac{\partial C}{\partial w_{37}}\right) = \left(\frac{\partial C}{\partial a_9}\right)\left(\frac{\partial a_9}{\partial z_9}\right)\left(\frac{\partial z_9}{\partial w_{37}}\right) \quad \text{where } \left(\frac{\partial z_9}{\partial w_{37}}\right) = a_2$$ Eqn 6.4c

$$\left(\frac{\partial C}{\partial w_{38}}\right) = \left(\frac{\partial C}{\partial a_9}\right)\left(\frac{\partial a_9}{\partial z_9}\right)\left(\frac{\partial z_9}{\partial w_{38}}\right) \quad \text{where } \left(\frac{\partial z_9}{\partial w_{38}}\right) = a_3$$ Eqn 6.4d

$$\left(\frac{\partial C}{\partial w_{39}}\right) = \left(\frac{\partial C}{\partial a_9}\right)\left(\frac{\partial a_9}{\partial z_9}\right)\left(\frac{\partial z_9}{\partial w_{39}}\right) \quad \text{where } \left(\frac{\partial z_9}{\partial w_{39}}\right) = a_4$$ Eqn 6.4e

$$\left(\frac{\partial C}{\partial w_{40}}\right) = \left(\frac{\partial C}{\partial a_9}\right)\left(\frac{\partial a_9}{\partial z_9}\right)\left(\frac{\partial z_9}{\partial w_{40}}\right) \quad \text{where } \left(\frac{\partial z_9}{\partial w_{40}}\right) = a_5$$ Eqn 6.4f

Hence: $\Delta b_9 = -L\left(\sum_{i=1}^{n}\left(\frac{\partial C}{\partial b_9}\right)\right)/n \quad \& \; b_{9(New)} = b_{9(Old)} + \Delta b_9$ Eqn 6.5a

$$\Delta w_{36} = -L\left(\sum_{i=1}^{n}\left(\frac{\partial C}{\partial w_{36}}\right)\right)/n \quad \& \; w_{36(New)} = w_{36(Old)} + \Delta w_{36}$$ Eqn 6.5b

$$\Delta w_{37} = -L\left(\sum_{i=1}^{n}\left(\frac{\partial C}{\partial w_{37}}\right)\right)/n \quad \& \; w_{37(New)} = w_{37(Old)} + \Delta w_{37}$$ Eqn 6.5c

$$\Delta w_{38} = -L\left(\sum_{i=1}^{n}\left(\frac{\partial C}{\partial w_{38}}\right)\right)/n \quad \& \; w_{38(New)} = w_{38(Old)} + \Delta w_{38}$$ Eqn 6.5d

$$\Delta w_{39} = -L\left(\sum_{i=1}^{n}\left(\frac{\partial C}{\partial w_{39}}\right)\right)/n \quad \& \; w_{39(New)} = w_{39(Old)} + \Delta w_{39}$$ Eqn 6.5e

$$\Delta w_{40} = -L\left(\sum_{i=1}^{n}\left(\frac{\partial C}{\partial w_{40}}\right)\right)/n \quad \& \; w_{40(New)} = w_{40(Old)} + \Delta w_{40}$$ Eqn 6.5f

$$\left(\frac{\partial C}{\partial b_{10}}\right) = \left(\frac{\partial C}{\partial a_{10}}\right)\left(\frac{\partial a_{10}}{\partial z_{10}}\right)\left(\frac{\partial z_{10}}{\partial b_{10}}\right) \quad \text{where } \left(\frac{\partial z_{10}}{\partial b_{10}}\right) = 1$$ Eqn 6.6a

$$\left(\frac{\partial C}{\partial w_{41}}\right) = \left(\frac{\partial C}{\partial a_{10}}\right)\left(\frac{\partial a_{10}}{\partial z_{10}}\right)\left(\frac{\partial z_{10}}{\partial w_{41}}\right) \quad \text{where } \left(\frac{\partial z_{10}}{\partial w_{41}}\right) = a_1 \; \& \; \left(\frac{\partial a_{10}}{\partial z_{10}}\right) = \sigma'(z_{10}) = \frac{e^{z_{10}}}{(1+e^{z_{10}})^2}$$ Eqn 6.6b

$$\left(\frac{\partial C}{\partial w_{42}}\right) = \left(\frac{\partial C}{\partial a_{10}}\right)\left(\frac{\partial a_{10}}{\partial z_{10}}\right)\left(\frac{\partial z_{10}}{\partial w_{42}}\right) \quad \text{where } \left(\frac{\partial z_{10}}{\partial w_{42}}\right) = a_2 \qquad \text{Eqn 6.6c}$$

$$\left(\frac{\partial C}{\partial w_{43}}\right) = \left(\frac{\partial C}{\partial a_{10}}\right)\left(\frac{\partial a_{10}}{\partial z_{10}}\right)\left(\frac{\partial z_{10}}{\partial w_{43}}\right) \quad \text{where } \left(\frac{\partial z_{10}}{\partial w_{43}}\right) = a_3 \qquad \text{Eqn 6.6d}$$

$$\left(\frac{\partial C}{\partial w_{44}}\right) = \left(\frac{\partial C}{\partial a_{10}}\right)\left(\frac{\partial a_{10}}{\partial z_{10}}\right)\left(\frac{\partial z_{10}}{\partial w_{44}}\right) \quad \text{where } \left(\frac{\partial z_{10}}{\partial w_{44}}\right) = a_4 \qquad \text{Eqn 6.6e}$$

$$\left(\frac{\partial C}{\partial w_{45}}\right) = \left(\frac{\partial C}{\partial a_{10}}\right)\left(\frac{\partial a_{10}}{\partial z_{10}}\right)\left(\frac{\partial z_{10}}{\partial w_{45}}\right) \quad \text{where } \left(\frac{\partial z_{10}}{\partial w_{45}}\right) = a_5 \qquad \text{Eqn 6.6f}$$

Hence: $\Delta b_{10} = -L\left(\sum_{i=1}^{n}\left(\frac{\partial C}{\partial b_{10}}\right)\right)/n$ & $b_{10(New)} = b_{10(Old)} + \Delta b_{10}$ Eqn 6.6a

$$\Delta w_{41} = -L\left(\sum_{i=1}^{n}\left(\frac{\partial C}{\partial w_{41}}\right)\right)/n \quad \& \quad w_{41(New)} = w_{41(Old)} + \Delta w_{41} \qquad \text{Eqn 6.6b}$$

$$\Delta w_{42} = -L\left(\sum_{i=1}^{n}\left(\frac{\partial C}{\partial w_{42}}\right)\right)/n \quad \& \quad w_{42(New)} = w_{42(Old)} + \Delta w_{42} \qquad \text{Eqn 6.6c}$$

$$\Delta w_{43} = -L\left(\sum_{i=1}^{n}\left(\frac{\partial C}{\partial w_{43}}\right)\right)/n \quad \& \quad w_{43(New)} = w_{43(Old)} + \Delta w_{43} \qquad \text{Eqn 6.6d}$$

$$\Delta w_{44} = -L\left(\sum_{i=1}^{n}\left(\frac{\partial C}{\partial w_{44}}\right)\right)/n \quad \& \quad w_{44(New)} = w_{44(Old)} + \Delta w_{44} \qquad \text{Eqn 6.6e}$$

$$\Delta w_{45} = -L\left(\sum_{i=1}^{n}\left(\frac{\partial C}{\partial w_{45}}\right)\right)/n \quad \& \quad w_{45(New)} = w_{45(Old)} + \Delta w_{45} \qquad \text{Eqn 6.6f}$$

3. The overall cost function on hidden neurons (HNs) $a_1 - a_5$.

This stage of mathematical formulation requires setting up the overall cost function of the selected hidden neurons ($a_1 - a_5$) against the cost function of the preceding activation (a_i), cost function and sum weights. For instance: $\left(\frac{\partial C}{\partial a_c}\right) = \left(\frac{\partial C}{\partial a_i}\right)\left(\frac{\partial a_i}{\partial z}\right)\left(\frac{\partial z}{\partial a_c}\right)$ where $w_c = \left(\frac{\partial z}{\partial a_c}\right)$ Eqn 6.7

Thus, the following mathematical models are obtained from the application of Eqn 6.7 to the DNN framework, that is Fig 2 in the main article.

$$\left(\frac{\partial C}{\partial a_1}\right)_1 = \left(\frac{\partial C}{\partial a_6}\right)\left(\frac{\partial a_6}{\partial z_6}\right)\left(\frac{\partial z_6}{\partial a_1}\right) \quad \text{where } \left(\frac{\partial a_6}{\partial z_6}\right) = \sigma'(z_6) = \frac{e^{z_6}}{(1+e^{z_6})^2} \;\&\; \left(\frac{\partial z_6}{\partial a_1}\right) = w_{21} \qquad \text{Eqn 6.8a}$$

$$\left(\frac{\partial C}{\partial a_1}\right)_2 = \left(\frac{\partial C}{\partial a_7}\right)\left(\frac{\partial a_7}{\partial z_7}\right)\left(\frac{\partial z_7}{\partial a_1}\right) \quad \text{where } \left(\frac{\partial a_7}{\partial z_7}\right) = \sigma'(z_7) = \frac{e^{z_7}}{(1+e^{z_7})^2} \;\&\; \left(\frac{\partial z_7}{\partial a_1}\right) = w_{26} \qquad \text{Eqn 6.8b}$$

$$\left(\frac{\partial C}{\partial a_1}\right)_3 = \left(\frac{\partial C}{\partial a_8}\right)\left(\frac{\partial a_8}{\partial z_8}\right)\left(\frac{\partial z_8}{\partial a_1}\right) \quad \text{where } \left(\frac{\partial a_8}{\partial z_8}\right) = \sigma'(z_8) = \frac{e^{z_8}}{(1+e^{z_8})^2} \;\&\; \left(\frac{\partial z_8}{\partial a_1}\right) = w_{31} \qquad \text{Eqn 6.8c}$$

$$\left(\frac{\partial C}{\partial a_1}\right)_4 = \left(\frac{\partial C}{\partial a_9}\right)\left(\frac{\partial a_9}{\partial z_9}\right)\left(\frac{\partial z_9}{\partial a_1}\right) \quad \text{where } \left(\frac{\partial a_9}{\partial z_9}\right) = \sigma'(z_9) = \frac{e^{z_9}}{(1+e^{z_9})^2} \;\&\; \left(\frac{\partial z_9}{\partial a_1}\right) = w_{36} \qquad \text{Eqn 6.8d}$$

$$\left(\frac{\partial C}{\partial a_1}\right)_5 = \left(\frac{\partial C}{\partial a_{10}}\right)\left(\frac{\partial a_{10}}{\partial z_{10}}\right)\left(\frac{\partial z_{10}}{\partial a_1}\right) \quad \text{where } \left(\frac{\partial a_{10}}{\partial z_{10}}\right) = \sigma'(z_{10}) = \frac{e^{z_{10}}}{(1+e^{z_{10}})^2} \;\&\; \left(\frac{\partial z_{10}}{\partial a_1}\right) = w_{41} \qquad \text{Eqn 6.8e}$$

$$\left(\frac{\partial C}{\partial a_1}\right) = \left(\frac{\partial C}{\partial a_1}\right)_1 + \left(\frac{\partial C}{\partial a_1}\right)_2 + \left(\frac{\partial C}{\partial a_1}\right)_3 + \left(\frac{\partial C}{\partial a_1}\right)_4 + \left(\frac{\partial C}{\partial a_1}\right)_5$$ Eqn 6.8f

$$\left(\frac{\partial C}{\partial a_2}\right)_1 = \left(\frac{\partial C}{\partial a_6}\right)\left(\frac{\partial a_6}{\partial z_6}\right)\left(\frac{\partial z_6}{\partial a_2}\right) \text{ where } \left(\frac{\partial a_6}{\partial z_6}\right) = \sigma'(z_6) = \frac{e^{z_6}}{(1+e^{z_6})^2} \ \& \left(\frac{\partial z_6}{\partial a_2}\right) = w_{22}$$ Eqn 6.9a

$$\left(\frac{\partial C}{\partial a_2}\right)_2 = \left(\frac{\partial C}{\partial a_7}\right)\left(\frac{\partial a_7}{\partial z_7}\right)\left(\frac{\partial z_7}{\partial a_2}\right) \text{ where } \left(\frac{\partial a_7}{\partial z_7}\right) = \sigma'(z_7) = \frac{e^{z_7}}{(1+e^{z_7})^2} \ \& \left(\frac{\partial z_7}{\partial a_2}\right) = w_{27}$$ Eqn 6.9b

$$\left(\frac{\partial C}{\partial a_2}\right)_3 = \left(\frac{\partial C}{\partial a_8}\right)\left(\frac{\partial a_8}{\partial z_8}\right)\left(\frac{\partial z_8}{\partial a_2}\right) \text{ where } \left(\frac{\partial a_8}{\partial z_8}\right) = \sigma'(z_8) = \frac{e^{z_8}}{(1+e^{z_8})^2} \ \& \left(\frac{\partial z_8}{\partial a_2}\right) = w_{32}$$ Eqn 6.9c

$$\left(\frac{\partial C}{\partial a_2}\right)_4 = \left(\frac{\partial C}{\partial a_9}\right)\left(\frac{\partial a_9}{\partial z_9}\right)\left(\frac{\partial z_9}{\partial a_2}\right) \text{ where } \left(\frac{\partial a_9}{\partial z_9}\right) = \sigma'(z_9) = \frac{e^{z_9}}{(1+e^{z_9})^2} \ \& \left(\frac{\partial z_9}{\partial a_2}\right) = w_{37}$$ Eqn 6.9d

$$\left(\frac{\partial C}{\partial a_2}\right)_5 = \left(\frac{\partial C}{\partial a_{10}}\right)\left(\frac{\partial a_{10}}{\partial z_{10}}\right)\left(\frac{\partial z_{10}}{\partial a_2}\right) \text{ where } \left(\frac{\partial a_{10}}{\partial z_{10}}\right) = \sigma'(z_{10}) = \frac{e^{z_{10}}}{(1+e^{z_{10}})^2} \ \& \left(\frac{\partial z_{10}}{\partial a_2}\right) = w_{42}$$ Eqn 6.9e

$$\left(\frac{\partial C}{\partial a_2}\right) = \left(\frac{\partial C}{\partial a_2}\right)_1 + \left(\frac{\partial C}{\partial a_2}\right)_2 + \left(\frac{\partial C}{\partial a_2}\right)_3 + \left(\frac{\partial C}{\partial a_2}\right)_4 + \left(\frac{\partial C}{\partial a_2}\right)_5$$ Eqn 6.9f

$$\left(\frac{\partial C}{\partial a_3}\right)_1 = \left(\frac{\partial C}{\partial a_6}\right)\left(\frac{\partial a_6}{\partial z_6}\right)\left(\frac{\partial z_6}{\partial a_3}\right) \text{ where } \left(\frac{\partial a_6}{\partial z_6}\right) = \sigma'(z_6) = \frac{e^{z_6}}{(1+e^{z_6})^2} \ \& \left(\frac{\partial z_6}{\partial a_3}\right) = w_{23}$$ Eqn 7.0a

$$\left(\frac{\partial C}{\partial a_3}\right)_2 = \left(\frac{\partial C}{\partial a_7}\right)\left(\frac{\partial a_7}{\partial z_7}\right)\left(\frac{\partial z_7}{\partial a_3}\right) \text{ where } \left(\frac{\partial a_7}{\partial z_7}\right) = \sigma'(z_7) = \frac{e^{z_7}}{(1+e^{z_7})^2} \ \& \left(\frac{\partial z_7}{\partial a_3}\right) = w_{28}$$ Eqn 7.0b

$$\left(\frac{\partial C}{\partial a_3}\right)_3 = \left(\frac{\partial C}{\partial a_8}\right)\left(\frac{\partial a_8}{\partial z_8}\right)\left(\frac{\partial z_8}{\partial a_3}\right) \text{ where } \left(\frac{\partial a_8}{\partial z_8}\right) = \sigma'(z_8) = \frac{e^{z_8}}{(1+e^{z_8})^2} \ \& \left(\frac{\partial z_8}{\partial a_3}\right) = w_{33}$$ Eqn 7.0c

$$\left(\frac{\partial C}{\partial a_3}\right)_4 = \left(\frac{\partial C}{\partial a_9}\right)\left(\frac{\partial a_9}{\partial z_9}\right)\left(\frac{\partial z_9}{\partial a_3}\right) \text{ where } \left(\frac{\partial a_9}{\partial z_9}\right) = \sigma'(z_9) = \frac{e^{z_9}}{(1+e^{z_9})^2} \ \& \left(\frac{\partial z_9}{\partial a_3}\right) = w_{38}$$ Eqn 7.0d

$$\left(\frac{\partial C}{\partial a_3}\right)_5 = \left(\frac{\partial C}{\partial a_{10}}\right)\left(\frac{\partial a_{10}}{\partial z_{10}}\right)\left(\frac{\partial z_{10}}{\partial a_3}\right) \text{ where } \left(\frac{\partial a_{10}}{\partial z_{10}}\right) = \sigma'(z_{10}) = \frac{e^{z_{10}}}{(1+e^{z_{10}})^2} \ \& \left(\frac{\partial z_{10}}{\partial a_3}\right) = w_{43}$$ Eqn 7.0e

$$\left(\frac{\partial C}{\partial a_3}\right) = \left(\frac{\partial C}{\partial a_3}\right)_1 + \left(\frac{\partial C}{\partial a_3}\right)_2 + \left(\frac{\partial C}{\partial a_3}\right)_3 + \left(\frac{\partial C}{\partial a_3}\right)_4 + \left(\frac{\partial C}{\partial a_3}\right)_5$$ Eqn 7.0f

$$\left(\frac{\partial C}{\partial a_4}\right)_1 = \left(\frac{\partial C}{\partial a_6}\right)\left(\frac{\partial a_6}{\partial z_6}\right)\left(\frac{\partial z_6}{\partial a_4}\right) \text{ where } \left(\frac{\partial a_6}{\partial z_6}\right) = \sigma'(z_6) = \frac{e^{z_6}}{(1+e^{z_6})^2} \ \& \left(\frac{\partial z_6}{\partial a_4}\right) = w_{24}$$ Eqn 7.1a

$$\left(\frac{\partial C}{\partial a_4}\right)_2 = \left(\frac{\partial C}{\partial a_7}\right)\left(\frac{\partial a_7}{\partial z_7}\right)\left(\frac{\partial z_7}{\partial a_4}\right) \text{ where } \left(\frac{\partial a_7}{\partial z_7}\right) = \sigma'(z_7) = \frac{e^{z_7}}{(1+e^{z_7})^2} \ \& \left(\frac{\partial z_7}{\partial a_4}\right) = w_{29}$$ Eqn 7.1b

$$\left(\frac{\partial C}{\partial a_4}\right)_3 = \left(\frac{\partial C}{\partial a_8}\right)\left(\frac{\partial a_8}{\partial z_8}\right)\left(\frac{\partial z_8}{\partial a_4}\right) \text{ where } \left(\frac{\partial a_8}{\partial z_8}\right) = \sigma'(z_8) = \frac{e^{z_8}}{(1+e^{z_8})^2} \ \& \left(\frac{\partial z_8}{\partial a_4}\right) = w_{34}$$ Eqn 7.1c

$$\left(\frac{\partial C}{\partial a_4}\right)_4 = \left(\frac{\partial C}{\partial a_9}\right)\left(\frac{\partial a_9}{\partial z_9}\right)\left(\frac{\partial z_9}{\partial a_4}\right) \text{ where } \left(\frac{\partial a_9}{\partial z_9}\right) = \sigma'(z_9) = \frac{e^{z_9}}{(1+e^{z_9})^2} \ \& \left(\frac{\partial z_9}{\partial a_4}\right) = w_{39}$$ Eqn 7.1d

$$\left(\frac{\partial C}{\partial a_4}\right)_5 = \left(\frac{\partial C}{\partial a_{10}}\right)\left(\frac{\partial a_{10}}{\partial z_{10}}\right)\left(\frac{\partial z_{10}}{\partial a_4}\right) \text{ where } \left(\frac{\partial a_{10}}{\partial z_{10}}\right) = \sigma'(z_{10}) = \frac{e^{z_{10}}}{(1+e^{z_{10}})^2} \ \& \left(\frac{\partial z_{10}}{\partial a_4}\right) = w_{44} \quad \text{Eqn 7.1e}$$

$$\left(\frac{\partial C}{\partial a_4}\right) = \left(\frac{\partial C}{\partial a_4}\right)_1 + \left(\frac{\partial C}{\partial a_4}\right)_2 + \left(\frac{\partial C}{\partial a_4}\right)_3 + \left(\frac{\partial C}{\partial a_4}\right)_4 + \left(\frac{\partial C}{\partial a_4}\right)_5 \quad \text{Eqn 7.1f}$$

$$\left(\frac{\partial C}{\partial a_5}\right)_1 = \left(\frac{\partial C}{\partial a_6}\right)\left(\frac{\partial a_6}{\partial z_6}\right)\left(\frac{\partial z_6}{\partial a_5}\right) \text{ where } \left(\frac{\partial a_6}{\partial z_6}\right) = \sigma'(z_6) = \frac{e^{z_6}}{(1+e^{z_6})^2} \ \& \left(\frac{\partial z_6}{\partial a_5}\right) = w_{25} \quad \text{Eqn 7.2a}$$

$$\left(\frac{\partial C}{\partial a_5}\right)_2 = \left(\frac{\partial C}{\partial a_7}\right)\left(\frac{\partial a_7}{\partial z_7}\right)\left(\frac{\partial z_7}{\partial a_5}\right) \text{ where } \left(\frac{\partial a_7}{\partial z_7}\right) = \sigma'(z_7) = \frac{e^{z_7}}{(1+e^{z_7})^2} \ \& \left(\frac{\partial z_7}{\partial a_5}\right) = w_{30} \quad \text{Eqn 7.2b}$$

$$\left(\frac{\partial C}{\partial a_5}\right)_3 = \left(\frac{\partial C}{\partial a_8}\right)\left(\frac{\partial a_8}{\partial z_8}\right)\left(\frac{\partial z_8}{\partial a_5}\right) \text{ where } \left(\frac{\partial a_8}{\partial z_8}\right) = \sigma'(z_8) = \frac{e^{z_8}}{(1+e^{z_8})^2} \ \& \left(\frac{\partial z_8}{\partial a_5}\right) = w_{35} \quad \text{Eqn 7.2c}$$

$$\left(\frac{\partial C}{\partial a_5}\right)_4 = \left(\frac{\partial C}{\partial a_9}\right)\left(\frac{\partial a_9}{\partial z_9}\right)\left(\frac{\partial z_9}{\partial a_5}\right) \text{ where } \left(\frac{\partial a_9}{\partial z_9}\right) = \sigma'(z_9) = \frac{e^{z_9}}{(1+e^{z_9})^2} \ \& \left(\frac{\partial z_9}{\partial a_5}\right) = w_{40} \quad \text{Eqn 7.2d}$$

$$\left(\frac{\partial C}{\partial a_5}\right)_5 = \left(\frac{\partial C}{\partial a_{10}}\right)\left(\frac{\partial a_{10}}{\partial z_{10}}\right)\left(\frac{\partial z_{10}}{\partial a_5}\right) \text{ where } \left(\frac{\partial a_{10}}{\partial z_{10}}\right) = \sigma'(z_{10}) = \frac{e^{z_{10}}}{(1+e^{z_{10}})^2} \ \& \left(\frac{\partial z_{10}}{\partial a_5}\right) = w_{45} \quad \text{Eqn 7.2e}$$

$$\left(\frac{\partial C}{\partial a_5}\right) = \left(\frac{\partial C}{\partial a_5}\right)_1 + \left(\frac{\partial C}{\partial a_5}\right)_2 + \left(\frac{\partial C}{\partial a_5}\right)_3 + \left(\frac{\partial C}{\partial a_5}\right)_4 + \left(\frac{\partial C}{\partial a_5}\right)_5 \quad \text{Eqn 7.2f}$$

4. The overall cost function on the input neurons $x_1 - x_4$.

This is the last stage of the cost optimisation techniques, and this approach requires going back to the familiar territory of adopting the single neural network of formulating mathematical models for the estimation of the changes in biases and weights connecting the input functions to the hidden neurons $a_1 - a_5$.

$$\left(\frac{\partial C}{\partial b_1}\right) = \left(\frac{\partial C}{\partial a_1}\right)\left(\frac{\partial a_1}{\partial z_1}\right)\left(\frac{\partial z_1}{\partial b_1}\right) \text{ where } \left(\frac{\partial z_1}{\partial b_1}\right) = 1 \quad \text{Eqn 7.3a}$$

$$\left(\frac{\partial C}{\partial w_1}\right) = \left(\frac{\partial C}{\partial a_1}\right)\left(\frac{\partial a_1}{\partial z_1}\right)\left(\frac{\partial z_1}{\partial w_1}\right) \text{ where } \left(\frac{\partial z_1}{\partial w_1}\right) = x_1 \ \& \left(\frac{\partial a_1}{\partial z_1}\right) = \sigma'(z_1) = \frac{e^{z_1}}{(1+e^{z_1})^2} \quad \text{Eqn 7.3b}$$

$$\left(\frac{\partial C}{\partial w_2}\right) = \left(\frac{\partial C}{\partial a_1}\right)\left(\frac{\partial a_1}{\partial z_1}\right)\left(\frac{\partial z_1}{\partial w_2}\right) \text{ where } \left(\frac{\partial z_1}{\partial w_2}\right) = x_2 \quad \text{Eqn 7.3c}$$

$$\left(\frac{\partial C}{\partial w_3}\right) = \left(\frac{\partial C}{\partial a_1}\right)\left(\frac{\partial a_1}{\partial z_1}\right)\left(\frac{\partial z_1}{\partial w_3}\right) \text{ where } \left(\frac{\partial z_1}{\partial w_3}\right) = x_3 \quad \text{Eqn 7.3d}$$

$$\left(\frac{\partial C}{\partial w_4}\right) = \left(\frac{\partial C}{\partial a_1}\right)\left(\frac{\partial a_1}{\partial z_1}\right)\left(\frac{\partial z_1}{\partial w_4}\right) \text{ where } \left(\frac{\partial z_1}{\partial w_4}\right) = x_4 \quad \text{Eqn 7.3e}$$

Hence: $\Delta b_1 = -L\left(\sum_{i=1}^{n}\left(\frac{\partial C}{\partial b_1}\right)\right)/n \ \& \ b_{1(Old)} = b_{1(New)} + \Delta b_1$ Eqn 7.4a

$\Delta w_1 = -L\left(\sum_{i=1}^{n}\left(\frac{\partial C}{\partial w_1}\right)\right)/n \ \& \ w_{1(New)} = w_{1(Old)} + \Delta w_1$ Eqn 7.4b

$\Delta w_2 = -L\left(\sum_{i=1}^{n}\left(\frac{\partial C}{\partial w_2}\right)\right)/n \ \& \ w_{2(New)} = w_{2(Old)} + \Delta w_2$ Eqn 7.4c

$$\Delta w_3 = -L\left(\sum_{i=1}^{n}\left(\frac{\partial C}{\partial w_3}\right)\right)/n \quad \& \; w_{3(New)} = w_{3(Old)} + \Delta w_3$$ Eqn 7.4d

$$\Delta w_4 = -L\left(\sum_{i=1}^{n}\left(\frac{\partial C}{\partial w_4}\right)\right)/n \quad \& \; w_{4(New)} = w_{4(Old)} + \Delta w_4$$ Eqn 7.4e

$$\left(\frac{\partial C}{\partial b_2}\right) = \left(\frac{\partial C}{\partial a_2}\right)\left(\frac{\partial a_2}{\partial z_2}\right)\left(\frac{\partial z_2}{\partial b_2}\right) \text{ where } \left(\frac{\partial z_2}{\partial b_2}\right) = 1$$ Eqn 7.5a

$$\left(\frac{\partial C}{\partial w_5}\right) = \left(\frac{\partial C}{\partial a_2}\right)\left(\frac{\partial a_2}{\partial z_2}\right)\left(\frac{\partial z_2}{\partial w_5}\right) \text{ where } \left(\frac{\partial z_2}{\partial w_5}\right) = x_1 \;\&\; \left(\frac{\partial a_2}{\partial z_2}\right) = \sigma'(z_2) = \frac{e^{z_2}}{(1+e^{z_2})^2}$$ Eqn 7.5b

$$\left(\frac{\partial C}{\partial w_6}\right) = \left(\frac{\partial C}{\partial a_2}\right)\left(\frac{\partial a_2}{\partial z_2}\right)\left(\frac{\partial z_2}{\partial w_6}\right) \text{ where } \left(\frac{\partial z_2}{\partial w_6}\right) = x_2$$ Eqn 7.5c

$$\left(\frac{\partial C}{\partial w_7}\right) = \left(\frac{\partial C}{\partial a_2}\right)\left(\frac{\partial a_2}{\partial z_2}\right)\left(\frac{\partial z_2}{\partial w_7}\right) \text{ where } \left(\frac{\partial z_2}{\partial w_7}\right) = x_3$$ Eqn 7.5d

$$\left(\frac{\partial C}{\partial w_8}\right) = \left(\frac{\partial C}{\partial a_2}\right)\left(\frac{\partial a_2}{\partial z_2}\right)\left(\frac{\partial z_2}{\partial w_8}\right) \text{ where } \left(\frac{\partial z_2}{\partial w_8}\right) = x_4$$ Eqn 7.5e

Hence: $\Delta b_2 = -L\left(\sum_{i=1}^{n}\left(\frac{\partial C}{\partial b_2}\right)\right)/n \quad \& \; b_{2(Old)} = b_{2(New)} + \Delta b_2$ Eqn 7.6a

$$\Delta w_5 = -L\left(\sum_{i=1}^{n}\left(\frac{\partial C}{\partial w_5}\right)\right)/n \quad \& \; w_{5(New)} = w_{5(Old)} + \Delta w_5$$ Eqn 7.6b

$$\Delta w_6 = -L\left(\sum_{i=1}^{n}\left(\frac{\partial C}{\partial w_6}\right)\right)/n \quad \& \; w_{6(New)} = w_{6(Old)} + \Delta w_6$$ Eqn 7.6c

$$\Delta w_7 = -L\left(\sum_{i=1}^{n}\left(\frac{\partial C}{\partial w_7}\right)\right)/n \quad \& \; w_{7(New)} = w_{7(Old)} + \Delta w_7$$ Eqn 7.6d

$$\Delta w_8 = -L\left(\sum_{i=1}^{n}\left(\frac{\partial C}{\partial w_8}\right)\right)/n \quad \& \; w_{8(New)} = w_{8(Old)} + \Delta w_8$$ Eqn 7.6e

$$\left(\frac{\partial C}{\partial b_3}\right) = \left(\frac{\partial C}{\partial a_3}\right)\left(\frac{\partial a_3}{\partial z_3}\right)\left(\frac{\partial z_3}{\partial b_3}\right) \text{ where } \left(\frac{\partial z_3}{\partial b_3}\right) = 1$$ Eqn 7.7a

$$\left(\frac{\partial C}{\partial w_9}\right) = \left(\frac{\partial C}{\partial a_3}\right)\left(\frac{\partial a_3}{\partial z_3}\right)\left(\frac{\partial z_3}{\partial w_9}\right) \text{ where } \left(\frac{\partial z_3}{\partial w_9}\right) = x_1 \;\&\; \left(\frac{\partial a_3}{\partial z_3}\right) = \sigma'(z_3) = \frac{e^{z_3}}{(1+e^{z_3})^2}$$ Eqn 7.7b

$$\left(\frac{\partial C}{\partial w_{10}}\right) = \left(\frac{\partial C}{\partial a_3}\right)\left(\frac{\partial a_3}{\partial z_3}\right)\left(\frac{\partial z_3}{\partial w_{10}}\right) \text{ where } \left(\frac{\partial z_3}{\partial w_{10}}\right) = x_2$$ Eqn 7.7c

$$\left(\frac{\partial C}{\partial w_{11}}\right) = \left(\frac{\partial C}{\partial a_3}\right)\left(\frac{\partial a_3}{\partial z_3}\right)\left(\frac{\partial z_3}{\partial w_{11}}\right) \text{ where } \left(\frac{\partial z_3}{\partial w_{11}}\right) = x_3$$ Eqn 7.7d

$$\left(\frac{\partial C}{\partial w_{12}}\right) = \left(\frac{\partial C}{\partial a_3}\right)\left(\frac{\partial a_3}{\partial z_3}\right)\left(\frac{\partial z_3}{\partial w_{12}}\right) \text{ where } \left(\frac{\partial z_3}{\partial w_{12}}\right) = x_4$$ Eqn 7.7e

Hence: $\Delta b_3 = -L\left(\sum_{i=1}^{n}\left(\frac{\partial C}{\partial b_3}\right)\right)/n \quad \& \; b_{3(Old)} = b_{3(New)} + \Delta b_3$ Eqn 7.7a

$\Delta w_9 = -L\left(\sum_{i=1}^{n}\left(\frac{\partial C}{\partial w_9}\right)\right)/n$ & $w_{9(New)} = w_{9(Old)} + \Delta w_9$ Eqn 7.7b

$\Delta w_{10} = -L\left(\sum_{i=1}^{n}\left(\frac{\partial C}{\partial w_{10}}\right)\right)/n$ & $w_{10(New)} = w_{10(Old)} + \Delta w_{10}$ Eqn 7.7c

$\Delta w_{11} = -L\left(\sum_{i=1}^{n}\left(\frac{\partial C}{\partial w_{11}}\right)\right)/n$ & $w_{11(New)} = w_{11(Old)} + \Delta w_{11}$ Eqn 7.7d

$\Delta w_{12} = -L\left(\sum_{i=1}^{n}\left(\frac{\partial C}{\partial w_{12}}\right)\right)/n$ & $w_{12(New)} = w_{12(Old)} + \Delta w_{12}$ Eqn 7.7e

$\left(\frac{\partial C}{\partial b_4}\right) = \left(\frac{\partial C}{\partial a_4}\right)\left(\frac{\partial a_4}{\partial z_4}\right)\left(\frac{\partial z_4}{\partial b_4}\right)$ where $\left(\frac{\partial z_4}{\partial b_4}\right) = 1$ Eqn 7.8a

$\left(\frac{\partial C}{\partial w_{13}}\right) = \left(\frac{\partial C}{\partial a_4}\right)\left(\frac{\partial a_4}{\partial z_4}\right)\left(\frac{\partial z_4}{\partial w_{13}}\right)$ where $\left(\frac{\partial z_4}{\partial w_{13}}\right) = x_1$ & $\left(\frac{\partial a_4}{\partial z_4}\right) = \sigma'(z_4) = \frac{e^{z_4}}{(1+e^{z_4})^2}$ Eqn 7.8b

$\left(\frac{\partial C}{\partial w_{14}}\right) = \left(\frac{\partial C}{\partial a_4}\right)\left(\frac{\partial a_4}{\partial z_4}\right)\left(\frac{\partial z_4}{\partial w_{14}}\right)$ where $\left(\frac{\partial z_4}{\partial w_{14}}\right) = x_2$ Eqn 7.8c

$\left(\frac{\partial C}{\partial w_{15}}\right) = \left(\frac{\partial C}{\partial a_4}\right)\left(\frac{\partial a_4}{\partial z_4}\right)\left(\frac{\partial z_4}{\partial w_{15}}\right)$ where $\left(\frac{\partial z_4}{\partial w_{15}}\right) = x_3$ Eqn 7.8d

$\left(\frac{\partial C}{\partial w_{16}}\right) = \left(\frac{\partial C}{\partial a_4}\right)\left(\frac{\partial a_4}{\partial z_4}\right)\left(\frac{\partial z_4}{\partial w_{16}}\right)$ where $\left(\frac{\partial z_4}{\partial w_{16}}\right) = x_4$ Eqn 7.8e

Hence: $\Delta b_4 = -L\left(\sum_{i=1}^{n}\left(\frac{\partial C}{\partial b_4}\right)\right)/n$ & $b_{4(Old)} = b_{4(New)} + \Delta b_4$ Eqn 7.8a

$\Delta w_{13} = -L\left(\sum_{i=1}^{n}\left(\frac{\partial C}{\partial w_{13}}\right)\right)/n$ & $w_{13(New)} = w_{13(Old)} + \Delta w_{13}$ Eqn 7.8b

$\Delta w_{14} = -L\left(\sum_{i=1}^{n}\left(\frac{\partial C}{\partial w_{14}}\right)\right)/n$ & $w_{14(New)} = w_{14(Old)} + \Delta w_{14}$ Eqn 7.8c

$\Delta w_{15} = -L\left(\sum_{i=1}^{n}\left(\frac{\partial C}{\partial w_{15}}\right)\right)/n$ & $w_{15(New)} = w_{15(Old)} + \Delta w_{15}$ Eqn 7.8d

$\Delta w_{16} = -L\left(\sum_{i=1}^{n}\left(\frac{\partial C}{\partial w_{16}}\right)\right)/n$ & $w_{16(New)} = w_{16(Old)} + \Delta w_{16}$ Eqn 7.8e

$\left(\frac{\partial C}{\partial b_5}\right) = \left(\frac{\partial C}{\partial a_5}\right)\left(\frac{\partial a_5}{\partial z_5}\right)\left(\frac{\partial z_5}{\partial b_5}\right)$ where $\left(\frac{\partial z_5}{\partial b_5}\right) = 1$ Eqn 8.0a

$\left(\frac{\partial C}{\partial w_{17}}\right) = \left(\frac{\partial C}{\partial a_5}\right)\left(\frac{\partial a_5}{\partial z_5}\right)\left(\frac{\partial z_5}{\partial w_{17}}\right)$ where $\left(\frac{\partial z_5}{\partial w_{17}}\right) = x_1$ & $\left(\frac{\partial a_5}{\partial z_5}\right) = \sigma'(z_5) = \frac{e^{z_5}}{(1+e^{z_5})^2}$ Eqn 8.0b

$\left(\frac{\partial C}{\partial w_{18}}\right) = \left(\frac{\partial C}{\partial a_5}\right)\left(\frac{\partial a_5}{\partial z_5}\right)\left(\frac{\partial z_5}{\partial w_{18}}\right)$ where $\left(\frac{\partial z_5}{\partial w_{18}}\right) = x_2$ Eqn 8.0c

$\left(\frac{\partial C}{\partial w_{19}}\right) = \left(\frac{\partial C}{\partial a_5}\right)\left(\frac{\partial a_5}{\partial z_5}\right)\left(\frac{\partial z_5}{\partial w_{19}}\right)$ where $\left(\frac{\partial z_5}{\partial w_{19}}\right) = x_3$ Eqn 8.0d

$$\left(\frac{\partial C}{\partial w_{20}}\right) = \left(\frac{\partial C}{\partial a_5}\right)\left(\frac{\partial a_5}{\partial z_5}\right)\left(\frac{\partial z_5}{\partial w_{20}}\right) \text{ where } \left(\frac{\partial z_5}{\partial w_{20}}\right) = x_4 \qquad \text{Eqn 8.0e}$$

Hence: $\Delta b_5 = -L\left(\sum_{i=1}^{n}\left(\frac{\partial C}{\partial b_5}\right)\right)/n$ & $b_{5(Old)} = b_{5(New)} + \Delta b_5$ Eqn 8.1a

$$\Delta w_{17} = -L\left(\sum_{i=1}^{n}\left(\frac{\partial C}{\partial w_{17}}\right)\right)/n \text{ \& } w_{17(New)} = w_{17(Old)} + \Delta w_{17} \qquad \text{Eqn 8.1b}$$

$$\Delta w_{18} = -L\left(\sum_{i=1}^{n}\left(\frac{\partial C}{\partial w_{18}}\right)\right)/n \text{ \& } w_{18(New)} = w_{18(Old)} + \Delta w_{18} \qquad \text{Eqn 8.1c}$$

$$\Delta w_{19} = -L\left(\sum_{i=1}^{n}\left(\frac{\partial C}{\partial w_{19}}\right)\right)/n \text{ \& } w_{19(New)} = w_{19(Old)} + \Delta w_{19} \qquad \text{Eqn 8.1d}$$

$$\Delta w_{20} = -L\left(\sum_{i=1}^{n}\left(\frac{\partial C}{\partial w_{20}}\right)\right)/n \text{ \& } w_{20(New)} = w_{20(Old)} + \Delta w_{20} \qquad \text{Eqn 8.1e}$$
